# Structured factorization for single-cell gene expression data

**DOI:** 10.1093/jrsssc/qlag009

**Published:** 2026-02-25

**Authors:** Antonio Canale, Luisa Galtarossa, Davide Risso, Lorenzo Schiavon, Giovanni Toto

**Affiliations:** Department of Statistical Sciences, University of Padova, 35121 Padova, Italy; Department of Statistical Sciences, University of Padova, 35121 Padova, Italy; Department of Statistical Sciences, University of Padova, 35121 Padova, Italy; Department of Statistical Sciences, University of Padova, 35121 Padova, Italy; Department of Statistics and Data Science, University of Texas at Austin, Austin, TX 78712, USA

## Abstract

Motivated by the analysis of complex single-cell gene expression data we propose a Bayesian class of generalized factor models for high dimensional count data. The developed methodology allows us to incorporate external knowledge, such as biological pathways, into the model’s prior distribution. This approach promotes sparsity in the factor loadings facilitating their interpretation and that of the corresponding latent factors. We demonstrate the effectiveness of our model on single-cell RNA sequencing data obtained from cord blood mononuclear cells, revealing promising insights into the role of pathways in characterizing gene relationships and extracting valuable information about unobserved cell traits.

## 1 Introduction

### 1.1 Single-cell RNA sequencing data

Single-cell RNA sequencing (scRNA-Seq) has become a widely used tool to characterize gene expression of thousands of cells at transcriptome-wide resolution. By sequencing RNA molecules from individual cells, scRNA-Seq provides a count-based measure of relative gene expression. Compared to previous ‘bulk’ technologies, single-cell sequencing unlocks the possibility to analyse rare cell types, to discover new cell types, and to study the heterogeneity of gene expression in cell populations of interest (Wagner et al., 2016). This is important in various fields, including cancer research, in which studying tumour samples at single-cell resolution allows for the discovery of cell sub-populations that potentially respond differently to treatment (Xue et al., 2020), and immunology, in which scRNA-Seq can be used to identify and characterize distinct immune cell types and to reconstruct developmental trajectories that reveal cell fate decisions of distinct cell subpopulations (Papalexi & Satija, 2018).

For each cell, scRNA-Seq data consist of counts that represent the expression of each gene in that cell. In a typical experiment, in addition to the cells by genes expression matrix, several supporting variables are collected for each of the analysed cells and for each of the measured genes; we name ‘covariates’ the former and ‘meta-covariates’ the latter.

We denote the matrix of gene expression as y; such matrix, of dimension n×p, contains the counts for p genes measured on n cells. The matrix containing the covariates, *x*, is a n×d matrix where *d* indicates the number of covariates for each cell. The covariates are cell-specific features, typically containing quality control information, such as the number of mapped or aligned reads and the total counts, as well as phenotypic information, such as the tissue or donor. The matrix containing the meta-covariates is indicated with *w*; it has dimensions p×q and contains the *q* meta-covariates for each gene, which are gene-specific features containing technical, e.g. gene length or GC content, and biological, e.g. pathway membership, information.

Gene expression data at single-cell resolution are highly informative, allowing researchers to characterize the cells at the finest level, and their application to cancer research, immunology, and developmental biology have already led to novel insights. However, scRNA-Seq data are challenging: they are high-dimensional count data, characterized by high variance and abundance of zeros. Hence, exploratory models are needed to facilitate summary, visualization and clustering of cells, to identify novel biological hypotheses to be tested with targeted experiments.

### 1.2 High dimensional count data challenges

A default strategy for modelling RNA-seq count data consists in using standard parametric distributions such as the Poisson (Marioni et al., 2008) or the negative binomial (Anders & Huber, 2010; Robinson & Smyth, 2008). Even if simple in terms of computation and interpretation, such standard models have some limitations. For instance, even the negative binomial may be unable to capture the zero inflation and multimodality of gene-wise distributions often observed in scRNA-Seq (Jiang et al., 2022).

A different, unfortunately still common, approach forgets the count nature of the data and treats them as continuous. A common practice, often used also in different applications in which count data are observed, consists of log- or square-root-transforming the observed counts, subsequently applying methods designed for continuous or Gaussian data. However, transformations to Gaussianity are ineffective for small counts (Warton, 2018), while log-transformations introduce difficulties in the presence of zeros (O’Hara & Kotze, 2010). This practice has been strongly criticized in our motivating context of scRNA-Seq data (e.g. Townes et al., 2019). More broadly, these approaches are not well-defined for count data: the data-generating process for a continuously-transformed Gaussian model cannot produce counts, which immediately amplifies model misspecification, limits interpretability, and undermines the reliability of inference and predictive distributions.

To address these challenges, we introduce a flexible Bayesian framework specifically tailored for complex count-valued data. The proposed approach relies on a continuous latent-variable representation, a strategy widely adopted in the Bayesian literature via data augmentation (Albert & Chib, 1993; Tanner & Wong, 1987). For count responses, related formulations have been developed by Canale and Dunson (2011) and Kowal and Canale (2020). By adopting this specification, we are able to effectively capture the various characteristics associated with high-dimensional count probability mass functions. In addition, to account for the intricate dependence structures present in the multivariate count vector, we follow the common practice that leverages factorization models. This approach allows us to express the high-dimensional covariance matrix as a combination of a limited number of rank-one matrices. Recently, Schiavon et al. (2022) introduced a general class of infinite factorization models capable of handling continuous, binary, and count data. Notably, this class of models promotes sparsity in the matrix of factor loadings by effectively incorporating information from covariate and meta-covariate vectors.

In the next section, we provide a detailed description of our proposed approach, which we refer to as cosin (COunt data Structured INfinite factorization). To assess the validity and generality of our approach, as well as to compare its performance against state-of-the-art scRNA-Seq methods, we present a comprehensive simulation experiment in Section 3. In Section 4, we apply this model to a dataset that characterizes the mononuclear cells of the cord blood. An additional application involving lung adenocarcinoma scRNA-Seq data is reported in the online supplementary material. Finally, in Section 5, we provide a thorough discussion of the proposed approach, its generalization and extensions, and its possible applications beyond scRNA-Seq studies.

## 2 Model and prior specification

For each cell i=1,…,n, scRNA-Seq data can be treated as a *p*-dimensional vector of integer valued random variables $y_{i}\in N^{p}$ where N is the set of natural numbers. Along with the n×p data matrix *y*, additional external information for each cell and each gene are also available. Without losing generality, let $x_{i}$ be the *d*-dimensional vector $\left(1,x_{i1},\ldots ,x_{id-1}\right)$, with first term equal to 1 and d−1 available cell-specific covariates. Let also $w_{j}$ for j=1,…,p be the *q*-dimensional vector of gene-specific meta-covariates with $w_{j}^{⊤}=(w_{Tj}^{⊤},w_{Bj}^{⊤})$ where $w_{Tj}$ is the $q_{T}$ dimensional subvector of technical meta-covariates with first element equal to 1, and $w_{Bj}$ is the $q_{B}$ dimensional subvector of biological meta-covariates such that $q=q_{T}+q_{B}$.

### 2.1 Model specification

Following Kowal and Canale (2020) we introduce a continuous-valued latent matrix *z* related to the observed count-valued matrix *y* via a simultaneous transformation and rounding operator S:R→N with S(⋅)=H(G(⋅)). Specifically, the rounding operator is such that H(t)=ℓ if $t\in A_{\ell }$ and $\{A_{\ell }{\}}_{\ell =0}^{\infty }$ is a known partition of R. Here, we adopt the floor function defined by $A_{\ell }=[\ell ,\ell +1)$. As discussed in Kowal and Canale (2020), rounding alone is suboptimal, particularly when the original data are counts. We specify the transformation operator G as the exponential transformation. Thus, the single entry $y_{ij}$ of *y* is linked to a latent $z_{ij}$ via the operator S and specifically $y_{ij}=\ell$ if $\exp\;\{z_{ij}\}\in [\ell ,\ell +1)$. While alternative link functions, including a nonparametric link as described by Kowal and Canale (2020), can be used, the proposed solution is the most straightforward choice, as it serves as the natural link for a Poisson GLM and aligns with common practice in the field while maintaining computational efficiency.

The latent variables $z_{ij}$ are modelled via


(1) $$z_{ij}=x_{i}^{T}\beta_{j}+\epsilon_{ij},$$

where $x_{i}^{T}\beta_{j}$ is the conditional expectation of $z_{ij}$, with $\beta_{j}$ a *d*-dimensional vectors of coefficients, and $\epsilon_{ij}$ is a zero-mean Gaussian error term. Note that we are assuming a linear relation between the mean of the latent variables and the set of cell-specific covariates $x_{i}$ and that this relation is changing with *j*, i.e. we assume that the same set of covariates may impact differently the different columns of the matrix *z*. Since $x_{i1}=1$ for any *i*, as specified above, $\beta_{\;j1}$ serves as the gene-specific intercept for gene *j*. Although we do not explicitly include a cell-specific intercept in our model, one could account for differences in total counts by standardizing the data per cell, as commonly done in the literature. Alternatively, the total count per cell can be included as an additional covariate, which is the approach preferred in this work.

The Gaussian error term captures all the residual variability not modelled by the linear predictor in (1). Consistently with this, we exploit a factor analytic representation that allows us to express $\epsilon_{i}$ as the linear combinations of latent *k*-dimensional factors $\eta_{i}$. More formally, we let


(2) $$\epsilon_{ij}=\sum_{h=1}^{k}\lambda_{\;jh}\eta_{ih}+\varepsilon_{ij},$$

where $\lambda_{\;jh}$ is an element of the p×k factor loadings matrix Λ and $\eta_{ih}$ is an element of the *h*th latent factor $\eta_{\cdot h}$, with h=1,…,k. The vectors $\varepsilon_{i}$ represent the remaining noise and are iid according to a *p*-variate Gaussian distribution N(0,Σ), with diagonal covariance matrix Σ. In matrix notation, the error matrix ϵ is equal to a sum of n×p rank-one matrices identified by the vector product $\eta_{\cdot h}\lambda_{\cdot h}^{⊤}$. We refer to such matrices as rank-one additive contributions $C_{h}$. Notably, if the number *k* of these contributions is k≤p, the factor representation leads to a parsimonious model.

### 2.2 Prior specification

Following a Bayesian approach we elicit suitable prior distributions for the model parameters. The conditional expectation of $z_{ij}$ is modelled through a linear combination of a vector of cell-specific covariates $x_{i}$ weighted by a regression parameter vector $\beta_{j}$, which differs over the genes j=1,…,p. The availability of gene-specific prior information $w_{j}$ allows one to model the regression parameters accordingly. Indeed, one may expect that the expression of a gene *j* in a certain cell *i* depends on the cell traits $x_{i}$, but with such relation varying according to the gene characteristics $w_{j}$. It is well known, for example, that the gene length and its sequence composition (e.g. the proportion of guanine and cytosine nucleotides, known as GC content) influence gene expression quantification, potentially in sample-specific ways (Love et al., 2016; Risso et al., 2011). Hence, we specify $\beta_{j}∼N_{d}(\Gamma_{T}w_{Tj},\sigma_{\beta }^{2}I_{d})$ where $\Gamma_{T}$ is a $d\times q_{T}$ coefficient matrix that model how the technical characteristics $w_{T}$ of the genes impact the cell quality control parameter. By leveraging the scale-location property of the Gaussian family, we can express $\beta_{j}=\Gamma_{T}w_{Tj}+\nu_{j}$, where $\nu_{j}∼N_{d}(0,\sigma_{\beta }^{2}I_{d})$. This formulation allows one to represent $z_{ij}$ by explicitly highlighting the direct additive contribution of the technical meta-covariates $w_{Tj}$. In multivariate regression, such hierarchical structure on the mean process is common when additional information on the column entities is available. For instance, one may expect that the impact of the number of mapped reads on the expression of gene *j* varies according to the gene’s technical traits. We set the prior of $\Gamma_{T}$ entries as independent standard Gaussian random variables.

Inspired by such a structure on the mean, we exploit the structured increasing shrinkage prior introduced by Schiavon et al. (2022) to induce a gene-specific effect also on the loadings Λ, which model the impact of the latent cell traits $\eta_{\cdot h}(h=1,\ldots ,k)$. Consistently, the variance of each loading element is decomposed through the product of a factor-specific scale $\theta_{h}$ and a local scale $\phi_{\;jh}$ leading to the following hierarchical prior


(3) $$\begin{array}{rl} \lambda_{\;jh} & ∼N(0,\theta_{h}\phi_{\;jh}),\;\theta_{h}=\vartheta_{h}\rho_{h},\\ \vartheta_{h}^{-1} & ∼\text{Ga}(a_{\theta },b_{\theta }),\;\rho_{h}∼\text{Ber}(1-\pi_{h}), \end{array}$$

where $\text{Ga}(a_{\theta },b_{\theta })$ indicates a gamma distribution with mean $a_{\theta }/b_{\theta }$ and variance $a_{\theta }/b_{\theta }^{2}$ and $\text{Ber}(1-\pi_{h})$ is a Bernoulli distribution with mean $1-\pi_{h}$.

In such construction, $\pi_{h}$ is the probability of factor *h* being shrunk to zero and is defined according to the stick-breaking construction


$$\pi_{h}=\sum_{l=1}^{h}u_{l},\;u_{l}=v_{l}\prod_{m=1}^{l-1}(1-v_{m}),\;v_{m}∼\text{Be}(1,\alpha ),$$

where Be(a,b) indicates the beta distribution with mean a/(a+b). Under this cumulative construction, $\pi_{h+1}>\pi_{h}$ for any h>0 and $\lim_{h\to \infty }\pi_{h}=1$ almost surely. The probability of being shrunk is increasing over the index *h* allowing for an infinite factorization model (Bhattacharya & Dunson, 2011) when *k* is set equal to +∞, which can be approximated by a truncated version of the same model. Legramanti et al. (2020), which firstly introduced the cumulative stick-breaking construction to define a class of infinite factor models, note that the prior expected number of non shrunk columns of Λ is $E(\sum_{h=1}^{\infty }\rho_{h})=\alpha$, suggesting setting *α* equal to the expected number of active latent factors.

The scale $\phi_{\;jh}$ has a Bernoulli prior distribution and regulates the local shrinkage of the loadings. We model this local behaviour assuming


$$E(\phi_{\;jh})=c_{p}{\text{logit}}^{-1}(w_{Bj}^{⊤}\gamma_{hB}),\;\gamma_{hB}∼N(0,\sigma_{\gamma }^{2}I_{q}),$$

where logit${-}^{1}(x)=e^{x}/(1+e^{x})$, $c_{p}\in (0,1)$ is a possible offset, and $\gamma_{hB}$ is the *h*th column vector of a $q_{B}\times k$ matrix $\Gamma_{B}$ with independent standard Gaussian prior. The vector $w_{Bj}$ represents the realization of $q_{B}$ available gene-specific meta-covariates that we think could influence the effect $\lambda_{jh}(h=1,\ldots ,k)$ of the latent unobserved covariates $\eta_{h}$, correspondingly to their role in the specification of the covariate effects *β*. Coefficients of the unobserved covariates are shrunk jointly in similar genes, i.e. with similar meta-covariates. In particular, we consider as meta-covariates the binary vector $w_{Bj}$ that indicates the biological pathways including gene *j*. We use pathways meta-covariates here, as we expect the factor loadings to be influenced by the biological processes that genes contribute to. In other words, we expect that genes that interact in a given biological process act in a coordinated way in defining the factors inferred by our model. We use pathway membership as a proxy for biological process, as usually done in bioinformatics (Khatri et al., 2012). In contrast to most recent literature on structured factorization (Heaps & Jermyn, 2024), we link the meta-covariates to the variance of the latent elements rather than their means. This approach has the advantage of accommodating also negative correlations among genes that interact within the same biological process. When considering pathway membership as meta-covariates, we account for the possible correlation among the expression of genes in the same pathway. However, the prior does not inform about the sign of this correlation, allowing some genes to be up-/down-regulated in response to the variation of other genes in the same pathway.

### 2.3 Posterior computation and point estimation

Posterior approximation is obtained through Markov chain Monte Carlo (MCMC) sampling. While MCMC provides full Bayesian inference, its main drawback is computational burden. As shown in Schiavon et al. (2024), when the number of meta covariates and factors *k* is small relative to *n* and *p*, the computational complexity of a single Gibbs iteration for the structured increasing shrinkage prior is $O(np(p^{2}+n^{2}))$. Faster alternatives exist for point-wise estimation: probabilistic matrix factorization (Mnih & Salakhutdinov, 2007) has complexity O(np(n+p)) per iteration, and the stagewise algorithm of Schiavon et al. (2024) achieves O(np), but neither yields full Bayesian uncertainty quantification. Mean-field variational approximation is another option given the availability of full conditionals, though it may struggle with multimodality, which is common in factor models. Given these limitations, we have chosen to retain MCMC, as it provides a well-balanced compromise between computational efficiency and accuracy.

Although our approach formally assumes an infinite number of latent factors, in practice we approximate the model by retaining only the set of *active* (i.e. non-negligible) factors. Following common practice in infinite factor models (Bhattacharya & Dunson, 2011; Legramanti et al., 2020; Schiavon et al., 2022), we use an adaptive Gibbs algorithm to infer the number of active factors while drawing from the posterior distribution. To ensure convergence of the Markov chain, as stated in Theorem 5 of Roberts and Rosenthal (2007), the value of the number of factors is adapted at certain iterations with exponentially decreasing probability.

At the adaptive iterations, active factors are identified as those characterized by non zero loadings column (i.e. $\rho_{h}=1$) and the redundant factors are discarded. If no redundant factors are identified, a new factor is activated, allowing the estimated number of active factors to grow without bound as needed. Given the number of factors *k* at a certain iteration, model parameters are drawn from the corresponding posterior full conditional distributions. Details are reported in Appendix A of the online supplementary material.

In Bayesian analysis, point-wise estimates are usually obtained by approximating the parameters’ posterior expectations via Monte Carlo averages over the samples drawn during the MCMC. However, it is well-known in the Bayesian factor model literature that the sample average cannot informatively summarize the posterior distribution of *Λ* and *η*, due to their nonidentifiability. In fact, both Λ and *η* are only identifiable up to an arbitrary *rotation P* with $PP^{⊤}=I_{k}$, causing sampling of such parameters from possibly different rotational alignments in different Gibbs iterations. Given the sign symmetry of possible rotations, Monte Carlo averages would result in poorly meaningful point-wise estimates around zero. On the other hand, the nonidentifiable possible rotations of the rank-one contributions $C_{h}=\eta_{\cdot h}\lambda_{\cdot h}^{⊤}$ are limited to the class of permutations of the indices. Then, focusing on rank-one contributions, identifiability is achieved by following the steps below.

- Order the contributions $C_{1}^{\left(T\right)},\ldots ,C_{k}^{\left(T\right)}$ sampled at the last iteration *T* of the Gibbs algorithm decreasingly with respect to the Frobenius norm.
- Use the re-ordered contributions of the last iteration $C_{1^{*}}^{\left(T\right)},\ldots ,C_{k^{*}}^{\left(T\right)}$ as a reference.
- For each Gibbs iteration t<T, re-order the contributions as follows. For $h^{*}=1,\ldots ,k$, the contribution $C_{h^{*}}$ corresponds to the non ordered Contribution $C_{h}$ with index
  $$h={\text{argmin}}_{l\in H_{h}}\|C_{h^{*}}^{\left(T\right)}-C_{l}^{\left(t\right)}{\|}_{F},$$
  where $H_{h}$ is the set of $k-h^{*}+1$ indices of non re-ordered contributions and $\|A{\|}_{F}$ denotes the Frobenius norm of matrix *A*.

To obtain point-wise estimates, we compute, for each h=1,…,k, the sample mean ${\bar{C}}_{h}=(\sum_{t=1}^{T}C_{h^{*}}^{\left(t\right)})/T$ over the re-ordered contributions. To investigate the behaviour of the factors scores *η*, we select a representative iteration of the Gibbs sampler by following the procedure described in Schiavon et al. (2022). For alternative postprocessing algorithms to align the samples of Λ or η we refer the reader to McParland et al. (2014), Aßmann et al. (2016) and Roy et al. (2021).

## 3 Simulation study

To illustrate the validity and generality of Cosin, we assess its performances through a simulation study. Our approach is compared against state-of-the-art dimensionality reduction techniques, namely Glm-pca (Townes et al., 2019), Newwave (Agostinis et al., 2022), principal component analysis (Pca) on log-transformed counts, Scgbm (Nicol & Miller, 2024), and Fast-glm-pca (Weine et al., 2024).

The empirical evaluation serves multiple purposes. First, we examine how well each method reconstructs the original underlying signal. Additionally, we assess their predictive performance in an out-of-sample setting.

To evaluate the ability of the different methods in reconstructing the original underlying signal, we proceed with two different strategies. The first investigates how effectively the estimated latent signal preserves the clustering of observations when synthetic data are generated under group structures. While many of the approaches are not explicitly designed for clustering, this comparison is a common practice in gene expression studies (Yeung & Ruzzo, 2001; Žurauskienė & Yau, 2016). We apply *K*-means clustering to the standardized first latent components and measure clustering performance using the Adjusted Rand Index (ARI).

We consider the following data generating process


$$y_{ij}=\lfloor \exp\;(z_{ij})\rfloor ,\;z_{ij}=C_{1ij}+C_{2ij}+C_{3ij}+\varepsilon_{ij},\;\varepsilon_{ij}∼N(0,\sigma^{2}).$$

Notably, this simulations settings produce zero inflated data, consistently with real scRNA-Seq data. To further mimic the situation observed in real data, we induce row-wise and column-wise sparsity over the contribution matrices $C_{h}$, with $C_{hij}=\eta_{ih}\lambda_{\;jh}$. In particular, we specify


$$\begin{array}{rl} \eta_{i1} & ∼N(\mu_{g1},1),\;\eta_{i3}∼N(\mu_{g3},1),\;\lambda_{\cdot 1},\lambda_{\cdot 2}∼N_{p}(0,I_{p}),\\ \eta_{i2} & ∼N(0,0.05^{2}),\;i>n/G,\;\eta_{l2}=1,\;l\leq n/G,\\ \lambda_{j3} & ∼N_{p}(0,0.05^{2}),\;j>p/2,\;\lambda_{m3}=1,\;m\leq p/2, \end{array}$$

where observation *i* belongs to group g=1,…,G, and the group means $\mu_{gh}$ are sampled from $N(0,3^{2})$. Sparsity in the rows and columns is respectively induced by $\eta_{\cdot 2}$ and $\lambda_{\cdot 3}$.

Twelve different scenarios are obtained varying the data dimensions (n,p) over the set {(50,100),  (200,100),(200,1000)}, idiosyncratic variance $\sigma^{2}$ over $\left\{0.1^{2},1\right\}$, and number of *groups G* over {5,10}. For each scenario, we simulate 50 synthetic data sets.

Figure 1 reports boxplots of ARI distributions across the replicated datasets. Cosin performs comparably to the best alternatives when σ=0.1, and outperforms all competitors in the noisier scenario of σ=1, particularly in high-dimensional settings. This superior performance may stem from the flexibility of Cosin, which does not require prespecification of the number of latent dimensions. Competing methods necessitate setting the number of latent factors *k*, which we define as the number of principal components required to explain 90% of the variability in log-transformed counts. In contrast, Cosin only requires an expected number of factors specified through the hyperparameter *α*. Even in the idealized scenario where the number of latent dimensions is correctly specified for all the methods (k=3 in our simulations), our approach remains highly competitive. Refer to Figure A1 in the online supplementary material and the comments therein.

**Figure 1.**
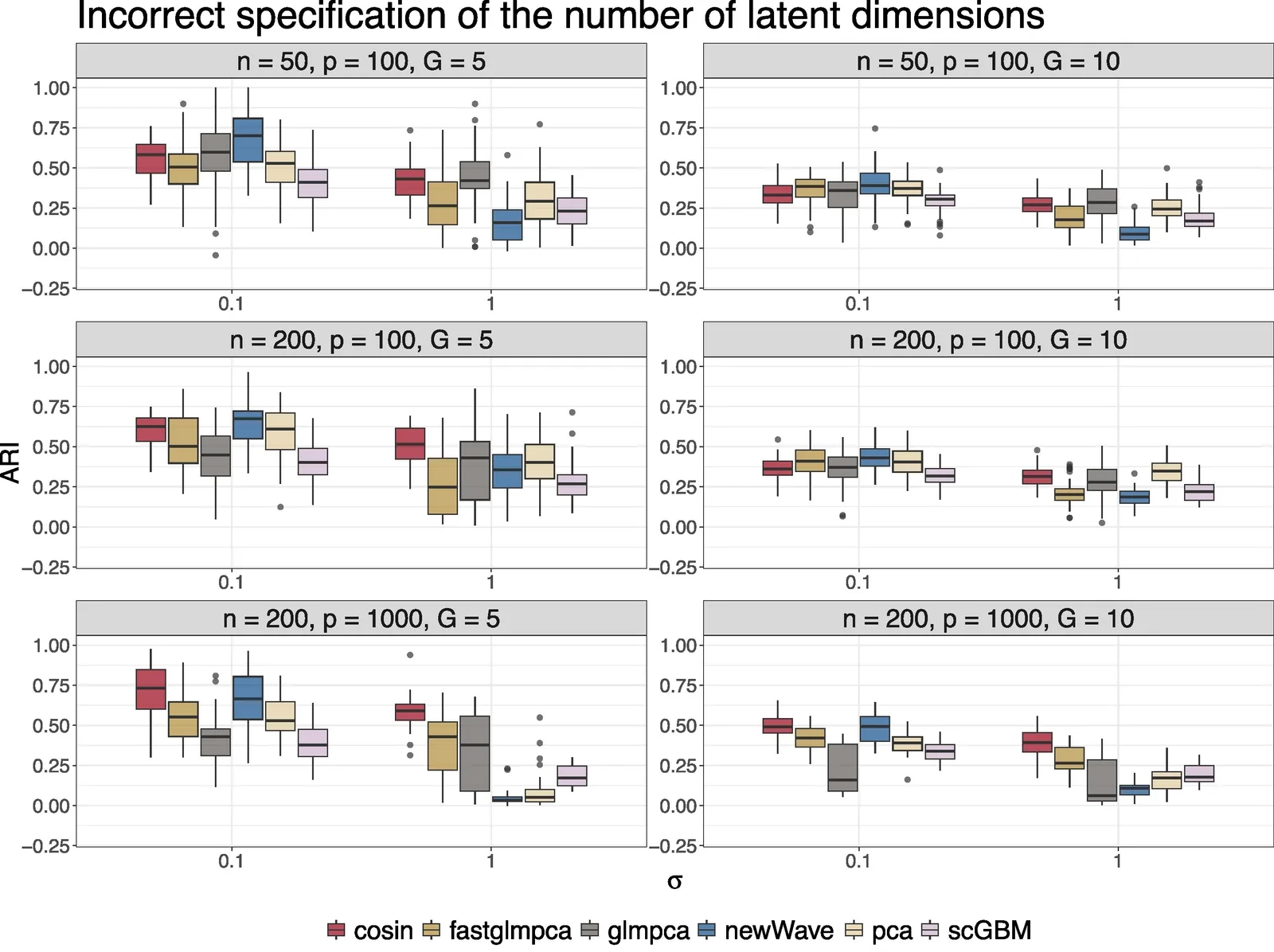
Boxplots of ARI of the competing models under different values of (n,p), $\sigma^{2}$ and *G*. Clustering is obtained applying a *G*-means clustering to the first *k* latent dimensions estimated by each method.

We now assess the ability of the models to estimate the original rank-one contributions noting that the rank-one contributions are identifiable under the conditions outlined in Section 2.3. The performances are measured in terms of root mean squared error (RMSE) of the entries of the contributions. To keep the discussion easier, we compare Cosin only with Glm-pca, the latter having a consistently stable and good performance in the previous experiment. Data are simulated from G=2 groups under the following specification


$$\begin{array}{rl} \eta_{\cdot 1},\eta_{\cdot 3} & ∼N_{n}(0,I_{n}),\;\lambda_{\cdot 1},\lambda_{\cdot 2}∼N_{p}(0,I_{p}),\\ \eta_{i2} & ∼N(0,0.05^{2}),\;i>n/2,\;\eta_{l2}=1,\;l\leq n/2,\\ \lambda_{j3} & ∼N_{p}(0,0.05^{2}),\;j>p/2,\;\lambda_{m3}=1,\;m\leq p/2, \end{array}$$

with different values of (n,p,σ) for 50 independent replicates.

Table 1 reports the Monte Carlo RMSE on the three contributions under the Cosin and Glm-pca models fitted with meta-covariates on the full data matrices. The results point out that the latent constructs identified by Glm-pca deviate from the contributions generating the data. This effect is particularly pronounced when Glm-pca overestimates the number of latent contributions, forcing the model to fit spurious factors to noise and leading to higher rates of false discoveries among the additional components. In contrast, Cosin retains consistently low errors even under over-specification, suggesting that it provides advantages in decoupling the multiple layers explaining residual variance in highly multivariate settings.

**Table 1.** Results of the simulation study for different values of (n,p,σ)

(n,p,σ)	Cosin				Glm-pca			
	$C_{1}$	$C_{2}$	$C_{3}$	*k*	$C_{1}$	$C_{2}$	$C_{3}$	*k*
(50,100,0.10)	0.63	0.58	0.39	3.29	1.85	1.76	0.84	8
	(0.25)	(0.28)	(0.26)	(1.00)	(2.13)	(1.25)	(0.18)	(1.00)
(50,100,1.00)	0.62	0.57	0.39	3	0.63	0.79	0.76	3
	(0.30)	(0.26)	(0.14)	(0.13)	(0.17)	(0.16)	(0.13)	(2.00)
(200,100,0.10)	0.54	0.45	0.25	3.11	1.56	1.44	0.74	8
	(0.35)	(0.33)	(0.19)	(0.34)	(0.58)	(0.78)	(0.03)	(0.75)
(200,100,1.00)	0.48	0.46	0.26	3	0.48	0.66	0.74	2
	(0.41)	(0.32)	(0.05)	(0.00)	(0.12)	(0.11)	(0.07)	(1.75)
(200,1000,0.10)	0.87	0.47	0.63	12.36	0.99	0.86	0.72	8
	(0.12)	(0.08)	(0.06)	(1.67)	(0.23)	(0.42)	(0.05)	(0.00)
(200,1000,1.00)	0.62	0.55	0.53	5	0.81	0.68	0.73	4
	(0.12)	(0.07)	(0.04)	(0.00)	(0.61)	(0.12)	(0.05)	(2.00)

*Note*. For the contributions $C_{h}$ the Monte Carlo RMSE is reported. For *k* the median of its estimates is reported. Interquartile ranges are reported in parenthesis.

Finally, to assess goodness-of-fit, we perform an out-of-sample prediction task, randomly removing a sample M of 25% of entries in *y*. Ignoring the entries of M, we fit Cosin, Cosin without meta-covariates, and Glm-pca. To assess the relative peformance in missing data imputation, we compute the mean absolute error of model *m* defined as


$${\text{MAE}}_{m}=\frac{1}{\left|M\right|}\sum_{l\in M}\left|y_{l}-{\hat{y}}_{l}^{\left(m\right)}\right|,$$

where ${\hat{y}}_{l}^{\left(m\right)}$ is the value predicted by the model *m*.

The true number of latent contributions is correctly identified by Cosin in over 80% of replications when p<1000 and is slightly overestimated in higher-dimensional settings. Importantly, this mild overestimation does not degrade performance, further supporting the conclusion that Cosin is less prone to spurious factor recovery and exhibits greater robustness to false discoveries. Furthermore, unlike our proposal, Glm-pca is not equipped of a methodology to infer the number of latent components k, which should be provided before the estimation. To favour a fair comparison, we estimate the Glm-pca models under different values of k∈{2,3,4,5,6,7,8}, using as a benchmark the specification characterized by the lowest MAE. Figure 2 provides a summary of the results for scenarios with $\sigma^{2}=1$. Our proposed approach shows the best performance across all scenarios. Although meta-covariates have a minor impact on model fitting, they play a crucial role in providing valuable and interpretable estimates, as will be clear in Section 4. The full results are presented in Table A1, reported in the online supplementary material.

**Figure 2.**
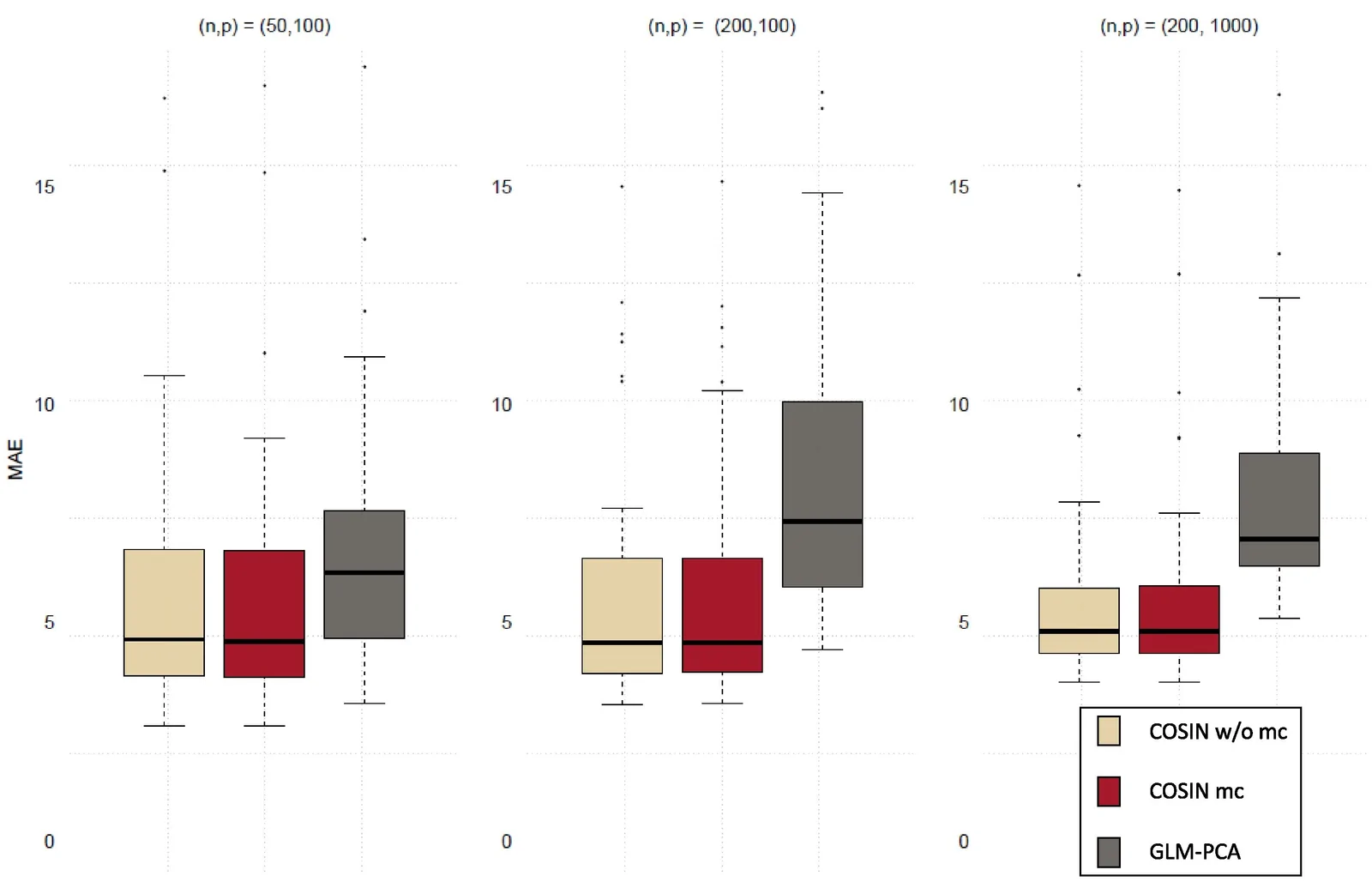
Boxplot of the out-of-sample MAE of the competing models under different values of (n,p) with $\sigma^{2}=1$. Since the R package glmpca does not allow for out-of-sample imputation, we adopt the Glm-pca approximation proposed by Townes et al. (2019)

## 4 CITE-Seq data

To show the utility of our approach in a real dataset, we reanalyse the cord blood mononuclear cells (CBMCs) CITE-seq dataset from Stoeckius et al. (2017). CITE-seq is a multi-modal protocol that allows for the simultaneous measurement of gene expression by scRNA-Seq in addition to the expression of several protein surface markers in the same cells. This is a key advantage in immunology studies, in which cell populations can be distinguished by the expression of such markers (Papalexi & Satija, 2018). Stoeckius et al. (2017) provide a set of rules to classify cells into cell types according to the expression of such markers (see their Figure 3).

**Figure 3.**
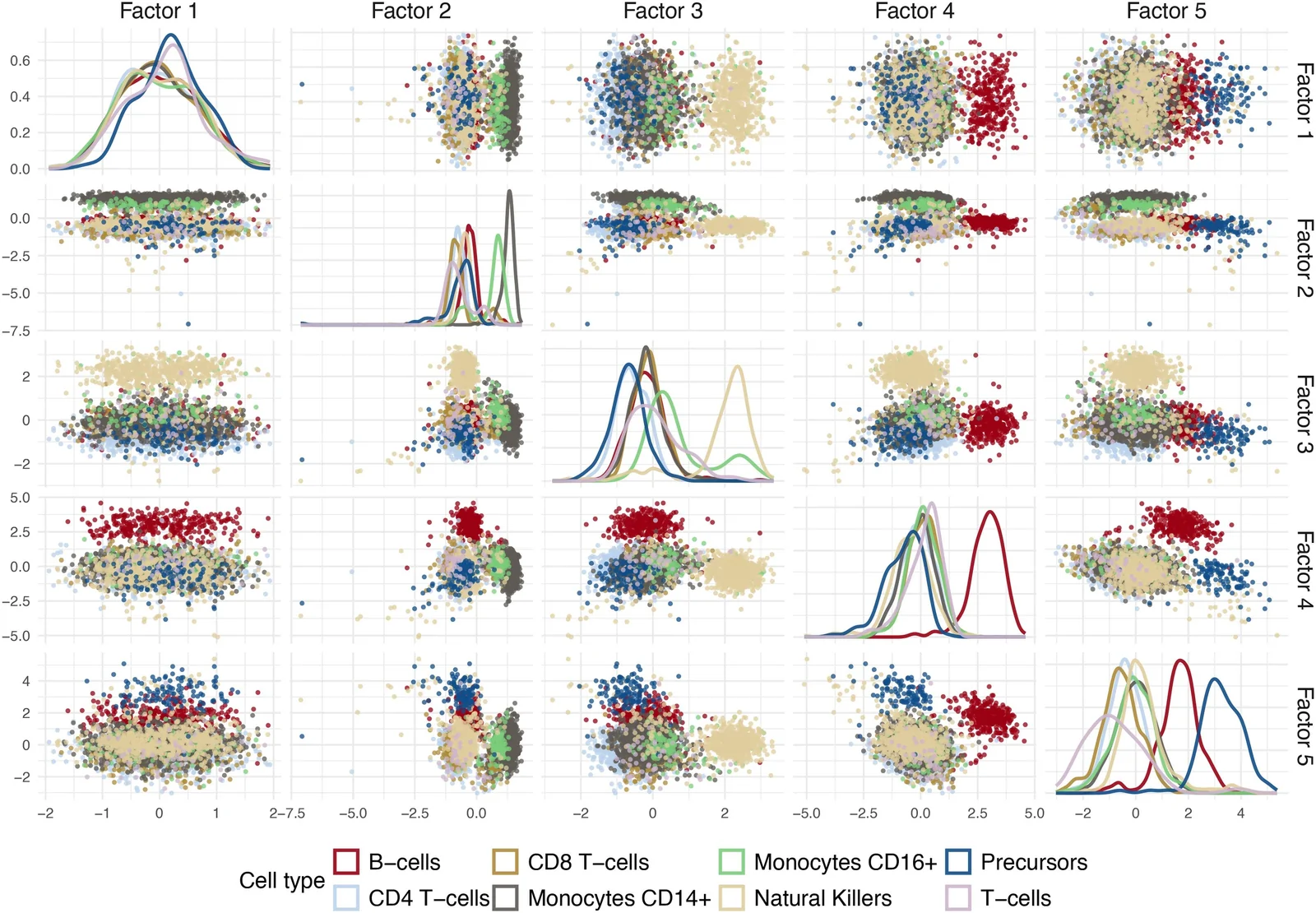
Density and pair plots of the scores of the first five latent factors of a representative posterior draw. Cell points are coloured according to the cell type.

We use the data provided in the *SingleCellMultimodal* Bioconductor package (Eckenrode et al., 2023), consisting of the expression of 36,280 genes in 7858 cells, along with a cell-type label based on the surface markers, which we consider as ground truth. We select the human genes available in the Ensembl database (v. 79) (Dyer et al., 2025) that are annotated as being part of the twenty immune system pathways in the KEGG database (Kanehisa et al., 2025) or in the remaining four largest pathways (see Table A2 in Appendix C of the online supplementary material). We further remove genes with missing information about length and GC content, and apply variance filtering to discard nonvariable genes.

The resulting data consist of a gene expression matrix of n=7858 cells and p=2572 genes. Along with this gene expression matrix, quality control information about the cells (covariates *x*) and the genes (technical meta-covariates $w_{Tj}$) are also available. The matrix *x* contains the number of expressed genes and the total number of counts. The inclusion of an intercept results in d=3. Moreover, cell type information is available; however, we chose not to include it as a covariate in the model. This decision reflects a common scenario in scRNA-Seq analysis, where cell identities are unknown beforehand. As a result, our setup provides a meaningful benchmark for evaluating the model’s ability to infer latent grouping structures in high-dimensional data. The technical meta-covariate matrix $w_{Tj}$ (j=1,…,p) includes gene-specific features such as length and GC content. In line with the motivations previously mentioned, for each gene *j*, we also define a biological meta-covariate binary vector $w_{Bj}$ with *m entry equal to 1 if the gene j belongs to the m* biological pathway. The list of $q_{B}=24$ pathways considered is provided in Table A2 of the online supplementary material.

We apply Cosin with the latent continuous Gaussian variable *z* specified as in equations (1)–(2). The prior distributions of the parameters follow the hierarchical structure described in Section 2. Given the high dimensionality of the dataset, we may expect a sufficiently large number of latent factors, hence we set the hyperparameter α=10 to control the number of nonshrunk contributions. To favour variable selection, we shrink covariate coefficients setting $\sigma_{\beta }^{2}=1/3$. After configuring the remaining hyperparameters in accordance with the simulation study, we run the algorithm for 15,000 iterations, discarding the first 5,000 as burn-in and saving every second sample to thin the Markov chain. Each Gibbs iteration required approximately three minutes using an Rcpp-based implementation on a machine equipped with an Intel(R) Xeon(R) Gold 6226R processor (2.90 GHz, 32 cores, 92 GB RAM). Although some autocorrelation is present, there are no evident convergence issues, as illustrated by the Markov chains shown in Figure A2 of the online supplementary material.

First, we briefly discuss the results obtained for the mean of the process and the cell covariate effects. Summaries of the covariates coefficient matrix *β* are reported in Table A3 in Appendix C of the online supplementary material. As expected, most of the gene-specific intercepts are strongly negative, reflecting the high probability of zero expression in the dataset. On the other hand, the number of detected genes per cell appears to be positively associated with gene expression. The effect of the total count per cell varies in direction across different genes, with part of this variability being explained by the technical characteristics of the genes, such as length and GC content. Indeed, as shown by the posterior distributions of the technical meta-covariates coefficients $\Gamma_{T}$ in Figure A3 of the online supplementary material, genes with high GC content and short length tend to exhibit higher expression, partly due to the positive impact of the total count per cell covariate. However, this effects are small, as expected in this dataset, which uses a protocol that only capture the $3^{\prime }$ end of the transcripts (Stoeckius et al., 2017), resulting in data less affected by length and GC content compared to bulk RNA-seq or SMART-seq2 data.

We focus the remainder of this section on the results obtained through the innovative treatment of the residual term enabled by Cosin. The adaptive Gibbs sampler identifies 31 active factors. While the dimensionality reduction is effective, the number of factors remains too high to analyse all of them in detail. Therefore, we focus on the five most relevant latent factors, specifically those with the highest Frobenius norm of the corresponding contribution matrices $C_{h}$ (h=1,…,31). While not discussed here, the further latent factors contribute to explaining the residual variance and may play a crucial role in guiding new biological discoveries in future studies.

To investigate the ability of the model to recognize potentially interpretable latent unobserved covariates, Figure 3 shows a representative posterior draw, selected according to the recommendations in Section 2.3, of the first five factor cell scores. Cells are coloured based on cell type to evaluate their correspondence with potential clusters revealed by our approach. We observe the orthogonality among the first factors and clear cell-type patterns, suggesting that the structure imposed on the latent part of the model aids in identifying and reconstructing possible missing covariates, with different factors distinguishing different cell types. The first factor is not correlated with any cell type or with observed cell-level covariates (see Figure A4 in the online supplementary material), suggesting that it may capture an unknown source of baseline cell heterogeneity. Performing unsupervised graph-based 8-group clustering with the Louvain algorithm on the scores of all 31 latent factors identifies clusters which show a high correspondence with the cell type groups, achieving an ARI of 0.757. The same clustering procedure was applied to the first 31 principal components identified by Glm-pca leading to an ARI of 0.689. Applying *k*-means clustering with k=8 to the standardized scores resulted in less consistency with the cell type groups with ARI of 0.446 and 0.551, respectively.

As discussed in Section 3, while incorporating exogenous information to inform the prior distribution of the loadings can aid in identifying the latent process and recognizing group structures among cells, this is not its primary objective. Rather, the use of biological pathway information is fundamental in providing a meaningful framework for interpreting the dependencies among genes induced by the loadings matrix. Figure 4 displays the gene-specific loading coefficients for the third and fourth factors of the representative posterior draw considered above. Directly below, bar plots of the corresponding sampled $\Gamma_{B}$ columns highlight the role of biological pathways in explaining the block of nonshrunk loadings entries. As evident in Figure 3, the scores of the third factor can be seen as the values of an unobserved covariate that differentiates *Natural Killer* cells. Consistently, in the top panel of Figure 4, we observe a block of nonshrunk loadings entries corresponding to the genes associated with the *Natural Killer cell-mediated cytotoxicity pathway*, which plays a key role in the immune function of these cells. Similarly, the fourth factor discriminate B-cells from the rest (Figure 3) and primarily explains variation in genes associated with the *Antigen processing and presentation pathway*, a key pathway distinguishing B-cells from other immune cell types (Figure 4). It is important to note that nonshrunk loadings explain both over-expression and under-expression of genes. For instance, B-cells and their precursors are characterized by larger scores in the fifth factor, which is characterized by the under-expression (negative loadings) of genes associated with the *T-cell receptor signalling pathway*, as shown in Figure A5 of the online supplementary material. This suggests that the model effectively captures biologically meaningful variation, uncovering latent structures that align with known immune cell functions.

**Figure 4.**
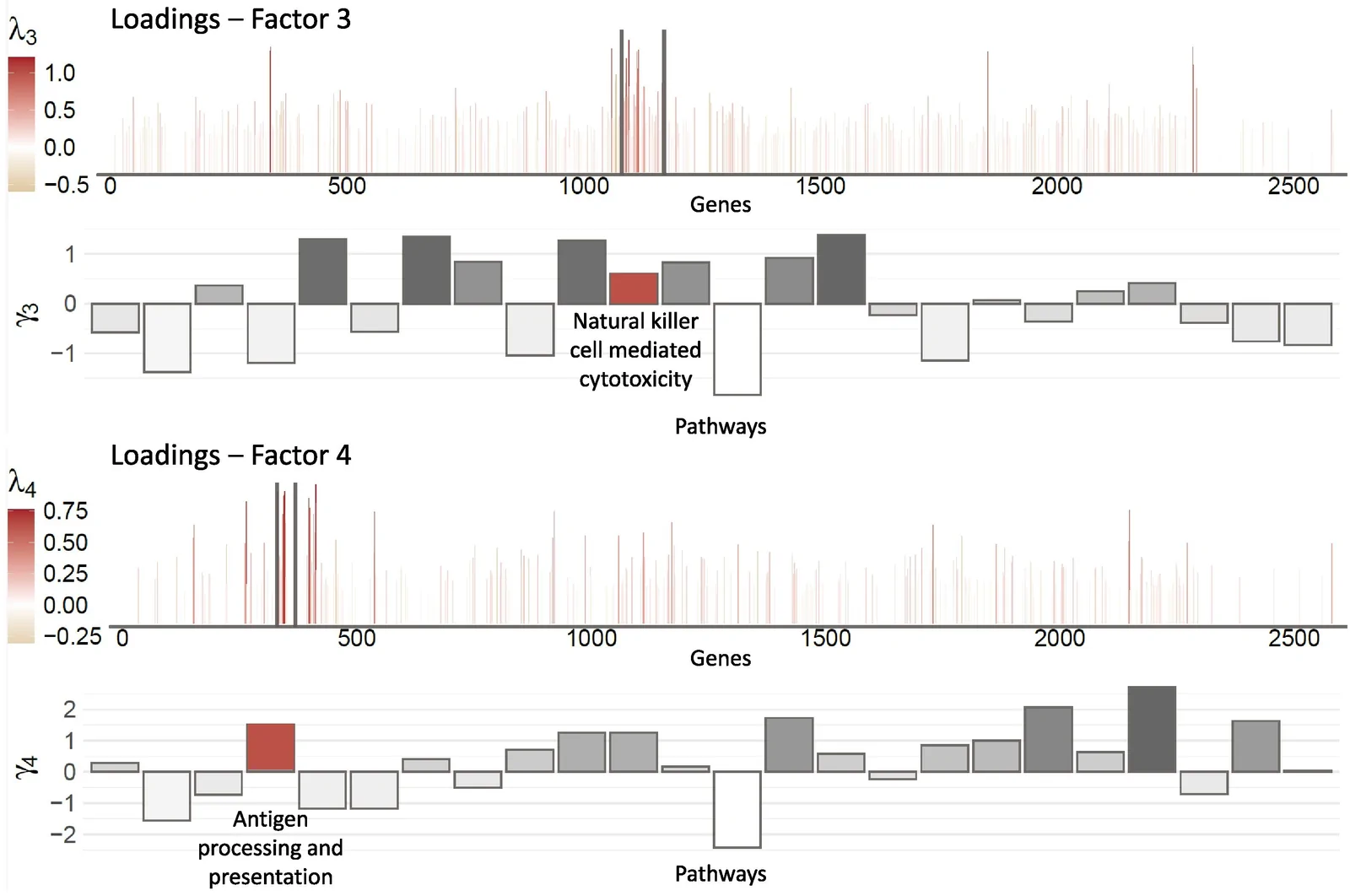
Barplots of the loadings and biological meta-covariate coefficients $\gamma_{B}$ of third and fourth factors of a representative posterior draw. The grey vertical lines on the loading barplots delimit the genes associated to the pathways highlighted in the corresponding *γ* barplots.

The multiplication of corresponding scores and loadings vectors results in rank-one factor contributions, which are identifiable quantities up to a possible reordering. After aligning their order in the posterior draws, we compute the mean of the 31 contribution matrices of dimension n×p, with the first five matrices displayed in Figures A6–A10 of the online supplementary material as level plots. The highlighted vertical blocks correspond to groups of genes associated with specific pathways, which often play a key role in these contributions to differentiate the cells according to their cell types (separated by horizontal lines in the plots).

However, not always the contributions that explain heterogeneity among cells are characterized by a differentiated impact on the genes with respect to the biological pathways. For instance, in the application of Cosin to lung adenocarcinoma cell data reported in the Appendix D of the online supplementary material, we observe a multiplicative factorization in different contributions of the cell type role and the genes pathway role in characterizing the gene expression.

Inducing structured sparsity in the loadings matrix Λ through the meta-covariate depending prior specified in Equation (3) favours the reconstruction of a sparse block structure in the covariance matrix of the underlying genes signal $var(z_{j})=\Lambda \Lambda^{⊤}+\Sigma$. To investigate the genes covariance structures, we reconstruct the undirected graph network based on the posterior mean of the partial correlation matrix of $z_{j}$. The graph, reported in Figure 5, reveals the presence of gene communities with genes belonging to the same pathway having the tendency of being clustered together. For instance, the cluster observed at the bottom of the graph is mainly constituted by the genes belonging to the *Chemokine signalling pathway*, while the genes related to the *Antigen processing and presentation pathway* are mainly distributed in the communities at the top of the graph. While this structure is favoured, yet not imposed, it facilitates a reconstruction of large covariance matrix, shrinking noninterpretable noise. In addition, the graph highlights the genes that stand out as hub nodes, since they are correlated with large groups of genes, or link different clusters. Notable hub genes include calcium binding proteins S100A8 and S100A9, associated with inflammation and immune response in the cord blood (Golubinskaya et al., 2020), and ALAS2 a gene essential in the development of red blood cells and whose mutation is the primal cause of congenital sideroblastic anaemia (Ducamp & Fleming, 2019). Other hub nodes include classic immune markers like PF4 (platelets), CD8B (cytotoxic T-cells), LILRA4 (dendritic cells), and HLA-DRA (B-cells, dendritic cells, and monocytes/macrophages) (Stelzer et al., 2016). Figure A11 in the online supplementary material reports the same graph but highlighting the positive and negative associations between genes.

**Figure 5.**
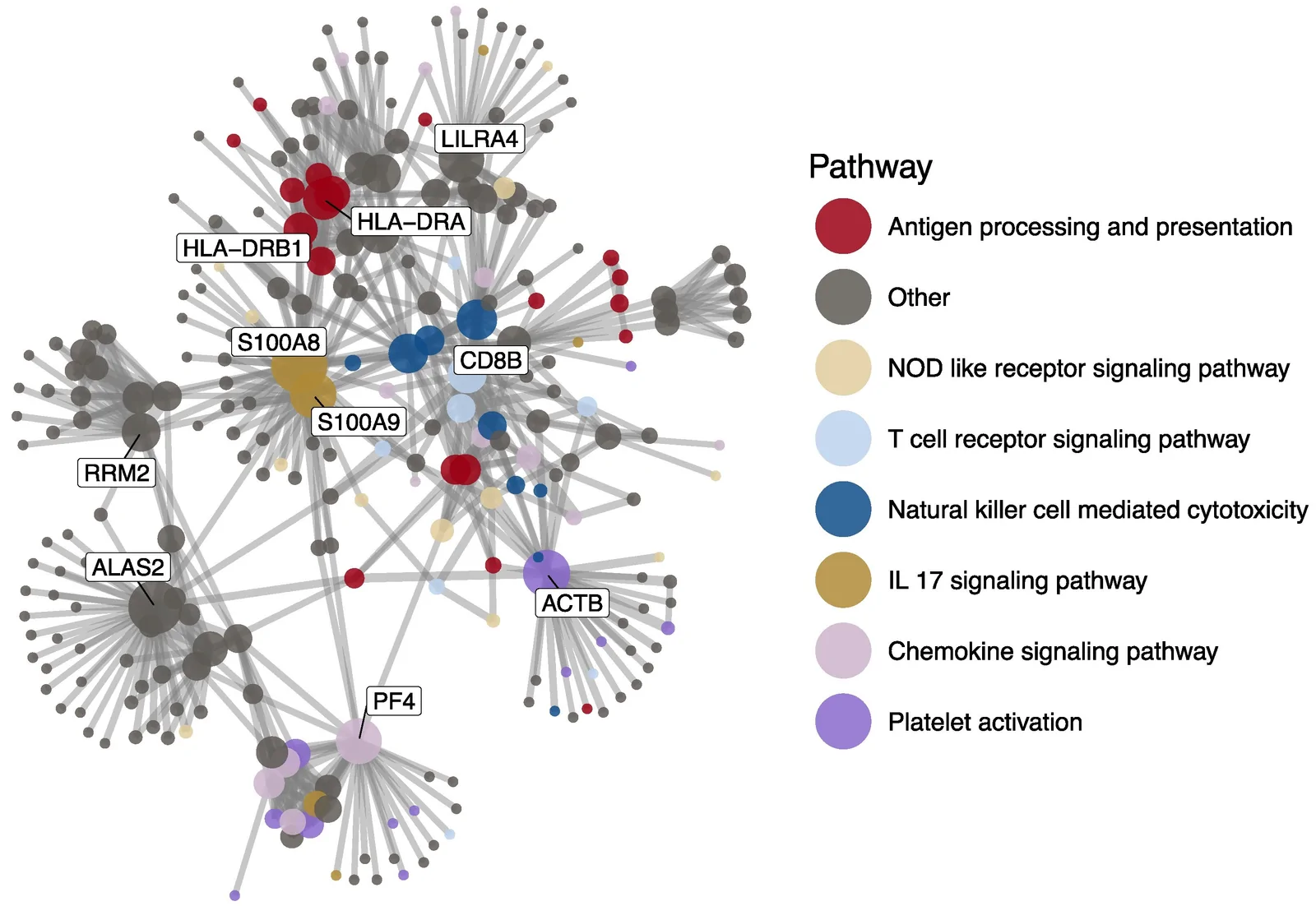
Graphical representation based on the posterior mean of the inverse of the correlation matrices estimated by the model. Edge thicknesses are proportional to the latent partial correlations between genes. Values below 0.025 are not reported. Nodes are positioned using a Fruchterman-Reingold force-direct algorithm and coloured according to the pathways the genes belong to.

## 5 Discussion

In this study, we introduced a novel method called Cosin which provides a joint modelling approach for multivariate count data through latent factor models. This approach was specifically motivated by scRNA-Seq applications, which involves complex count data. The empirical performances observed in real and synthetic data sets demonstrate that Cosin shows competitive results in terms of model fitting compared to existing methods, indicating its efficacy as a modelling framework.

One key advantage of Cosin is that it allows for the modelling of latent sparsity through the use of meta-covariates. While principally aimed at dimensionality reduction, it yields a well-structured latent space that readily supports subsequent cell clustering. A compelling direction for future work would be to incorporate Bayesian nonparametric mixture models to formalize this clustering process. Notably, Chandra et al. (2023) pioneered a joint inference framework that simultaneously learns the latent representation and the clustering assignment, a strategy that is especially advantageous in the high-dimensional context of single-cell RNA sequencing data. Further directions for future work include the development of more computationally efficient inference strategies, such as variational or stagewise approximations, to make full Bayesian inference practical for very large datasets, as well as the exploration of the proposed structured latent factor model under alternative likelihood specifications for count data, to further assess the relative role of the STAR construction and latent structure.

As discussed in Section 4, Cosin was successfully applied to CBMCs from the cord blood, where it discriminated the main immune cell types, highlighting the role of gene pathways in explaining the factors and identifying hub genes that may call attention to specific biological processes. These findings underline the potential utility of Cosin for uncovering biologically meaningful patterns in high-dimensional count data.

One important aspect to consider is the role of the intercept in the prior mean of the coefficients $\beta_{j}$. Indeed, this is a gene-wise intercept that helps the model accounts for the differences in sequencing depth across cells, similarly to what is achieved with the gene-wise intercept in Glm-pca (Townes et al., 2019) and with offsets in more traditional frequentist models (Love et al., 2014; Robinson et al., 2010).

Clearly, our results suggest that Cosin is applicable beyond genomics and can be used in any context dealing with complex high-dimensional counts. This versatility makes Cosin a valuable tool for researchers working in diverse fields.

## Supplementary Material

qlag009_Supplementary_Data

## Data Availability

The CITE-seq dataset is publicly available as part of the SingleCellMultiModal Bioconductor package, available at bioconductor.org/packages/SingleCellMultiModal. The lung adenocarcinoma cell line dataset is publicly available at github.com/LuyiTian/sc_mixology.

## References

[qlag009-B1] Agostinis F., Romualdi C., Sales G., & Risso D. (2022). Newwave: A scalable r/bioconductor package for the dimensionality reduction and batch effect removal of single-cell rna-seq data. Bioinformatics, 38(9), 2648–2650. 10.1093/bioinformatics/btac149 PMC9048694 35266509

[qlag009-B2] Albert J. H., & Chib S. (1993). Bayesian analysis of binary and polychotomous response data. Journal of the American Statistical Association, 88(422), 669–679. 10.1080/01621459.1993.10476321

[qlag009-B3] Anders S., & Huber W. (2010). Differential expression analysis for sequence count data. Nature Precedings, 11, 1–10. 10.1038/npre.2010.4282.1 PMC3218662 20979621

[qlag009-B4] Aßmann C., Boysen-Hogrefe J., & Pape M. (2016). Bayesian analysis of static and dynamic factor models: An ex-post approach towards the rotation problem. Journal of Econometrics, 192(1), 190–206. 10.1016/j.jeconom.2015.10.010

[qlag009-B5] Bhattacharya A., & Dunson D. B. (2011). Sparse bayesian infinite factor models. Biometrika, 98(2), 291–306. 10.1093/biomet/asr013 PMC3419391 23049129

[qlag009-B6] Canale A., & Dunson D. B. (2011). Bayesian kernel mixtures for counts. Journal of the American Statistical Association, 106(496), 1528–1539. 10.1198/jasa.2011.tm10552 PMC3329131 22523437

[qlag009-B7] Chandra N. K., Canale A., & Dunson D. B. (2023). Escaping the curse of dimensionality in Bayesian model-based clustering. Journal of Machine Learning Research, 24, 1–42. PMC11999651 40236516

[qlag009-B8] Ducamp S., & Fleming M. D. (2019). The molecular genetics of sideroblastic anemia. Blood, The Journal of the American Society of Hematology, 133, 59–69. 10.1182/blood-2018-08-815951 PMC6318428 30401706

[qlag009-B9] Dyer S. C., Austine-Orimoloye O., Azov A. G., Barba M., Barnes I., Barrera-Enriquez V. P., Becker A., Bennett R., Beracochea M., Berry A., Bhai J., Bhurji S. K., Boddu S., Lins P. R. B., Brooks L., Ramaraju S. B., Campbell L. I., Martinez M. C., Charkhchi M., …Yates A. D. (2025). Ensembl 2025. Nucleic Acids Research, 53(D1), D948–D957. 10.1093/nar/gkae1071 PMC11701638 39656687

[qlag009-B10] Eckenrode K. B., Righelli D., Ramos M., Argelaguet R., Vanderaa C., Geistlinger L., Culhane A. C., Gatto L., Carey V., Morgan M., Risso D., Waldron L., & Li M. (2023). Curated single cell multimodal landmark datasets for r/bioconductor. PLoS Computational Biology, 19(8), e1011324. 10.1371/journal.pcbi.1011324 PMC10497156 37624866

[qlag009-B11] Golubinskaya V., Puttonen H., Fyhr I.-M., Rydbeck H., Hellström A., Jacobsson B., Nilsson H., Mallard C., & Sävman K. (2020). Expression of s100a alarmins in cord blood monocytes is highly associated with chorioamnionitis and fetal inflammation in preterm infants. Frontiers in Immunology, 11, 1194. 10.3389/fimmu.2020.01194 PMC7308505 32612607

[qlag009-B12] Heaps S. E., & Jermyn I. H. (2024). Structured prior distributions for the covariance matrix in latent factor models. Statistics and Computing, 34(4), 1–18. 10.1007/s11222-024-10454-0.

[qlag009-B13] Jiang R., Sun T., Song D., & Li J. J. (2022). Statistics or biology: The zero-inflation controversy about scrna-seq data. Genome Biology, 23, 1–24. 10.1186/s13059-021-02568-9 PMC8783472 35063006

[qlag009-B14] Kanehisa M., Furumichi M., Sato Y., Matsuura Y., & Ishiguro-Watanabe M. (2025). Kegg: Biological systems database as a model of the real world. Nucleic Acids Research, 53(D1), D672–D677. 10.1093/nar/gkae909 PMC11701520 39417505

[qlag009-B15] Khatri P., Sirota M., & Butte A. J. (2012). Ten years of pathway analysis: Current approaches and outstanding challenges. PLoS Computational Biology, 8(2), e1002375. 10.1371/journal.pcbi.1002375 PMC3285573 22383865

[qlag009-B16] Kowal, D. R., & Canale, A. (2020). Simultaneous transformation and rounding (STAR) models for integer-valued data. Electronic Journal of Statistics, 14(1), 1744–1772. 10.1214/20-EJS1707.

[qlag009-B17] Legramanti S., Durante D., & Dunson D. B. (2020). Bayesian cumulative shrinkage for infinite factorizations. Biometrika, 107(3), 745–752. 10.1093/biomet/asaa008 PMC7430941 32831355

[qlag009-B18] Love M. I., Hogenesch J. B., & Irizarry R. A. (2016). Modeling of rna-seq fragment sequence bias reduces systematic errors in transcript abundance estimation. Nature Biotechnology, 34(12), 1287–1291. 10.1038/nbt.3682 PMC5143225 27669167

[qlag009-B19] Love M. I., Huber W., & Anders S. (2014). Moderated estimation of fold change and dispersion for rna-seq data with deseq2. Genome Biology, 15(12), 1–21. 10.1186/s13059-014-0550-8 PMC4302049 25516281

[qlag009-B20] Marioni J. C., Mason C. E., Mane S. M., Stephens M., & Gilad Y. (2008). Rna-seq: An assessment of technical reproducibility and comparison with gene expression arrays. Genome Research, 18(9), 1509–1517. 10.1101/gr.079558.108 PMC2527709 18550803

[qlag009-B21] McParland D., Gormley I. C., McCormick T. H., Clark S. J., Kabudula C. W., & Collinson M. A. (2014). Clustering south African households based on their asset status using latent variable models. The Annals of Applied Statistics, 8(2), 747. 10.1214/14-AOAS726 PMC4256055 25485026

[qlag009-B22] Mnih A., & Salakhutdinov R. R. (2007). Probabilistic matrix factorization. Advances in Neural Information Processing Systems, 20, 1–8.

[qlag009-B23] Nicol P. B., & Miller J. W. (2024). Model-based dimensionality reduction for single-cell rna-seq using generalized bilinear models. Biostatistics, 26(1), kxaf024. 10.1093/biostatistics/kxaf024 PMC12342792 40795862

[qlag009-B24] O’Hara R., & Kotze J. (2010). Do not log-transform count data. Nature Precedings, 1–17. 10.1038/npre.2010.4136.1

[qlag009-B25] Papalexi E., & Satija R. (2018). Single-cell rna sequencing to explore immune cell heterogeneity. Nature Reviews: Immunology, 18(1), 35–45. 10.1038/nri.2017.76 28787399

[qlag009-B26] Risso D., Schwartz K., Sherlock G., & Dudoit S. (2011). Gc-content normalization for rna-seq data. BMC Bioinformatics, 12(1), 1–17. 10.1186/1471-2105-12-480 PMC3315510 22177264

[qlag009-B27] Roberts G. O., & Rosenthal J. S. (2007). Coupling and ergodicity of adaptive markov chain monte carlo algorithms. Journal of Applied Probability, 44(2), 458–475. 10.1239/jap/1183667414

[qlag009-B28] Robinson M. D., McCarthy D. J., & Smyth G. K. (2010). edger: A bioconductor package for differential expression analysis of digital gene expression data. Bioinformatics, 26(1), 139–140. 10.1093/bioinformatics/btp616 PMC2796818 19910308

[qlag009-B29] Robinson M. D., & Smyth G. K. (2008). Small-sample estimation of negative binomial dispersion, with applications to sage data. Biostatistics, 9(2), 321–332. 10.1093/biostatistics/kxm030 17728317

[qlag009-B30] Roy A., Borg J. S., & Dunson D. B. (2021). Bayesian time-aligned factor analysis of paired multivariate time series. Journal of Machine Learning Research: JMLR, 22, 11347–11373. PMC9221555 35754922

[qlag009-B31] Schiavon L., Canale A., & Dunson D. B. (2022). Generalized infinite factorization models. Biometrika, 109(3), 817–835. 10.1093/biomet/asab056 PMC9469809 36105175

[qlag009-B32] Schiavon L., Nipoti B., & Canale A. (2024). Accelerated structured matrix factorization. Journal of Computational and Graphical Statistics, 33(3), 917–927. 10.1080/10618600.2023.2301072

[qlag009-B33] Stelzer G., Rosen N., Plaschkes I., Zimmerman S., Twik M., Fishilevich S., Stein T. I., Nudel R., Lieder I., Mazor Y., Kaplan S., Dahary D., Warshawsky D., Guan-Golan Y., Kohn A., Rappaport N., Safran M., & Lancet D. (2016). The genecards suite: From gene data mining to disease genome sequence analyses. Current Protocols in Bioinformatics, 54(1), 1–30. 10.1002/0471250953.2016.54.issue-1 27322403

[qlag009-B34] Stoeckius M., Hafemeister C., Stephenson W., Houck-Loomis B., Chattopadhyay P. K., Swerdlow H., Satija R., & Smibert P. (2017). Simultaneous epitope and transcriptome measurement in single cells. Nature Methods, 14(9), 865–868. 10.1038/nmeth.4380 PMC5669064 28759029

[qlag009-B35] Tanner M. A., & Wong W. H. (1987). The calculation of posterior distributions by data augmentation. Journal of the American Statistical Association, 82(398), 528–540. 10.1080/01621459.1987.10478458

[qlag009-B36] Townes F. W., Hicks S. C., Aryee M. J., & Irizarry R. A. (2019). Feature selection and dimension reduction for single-cell rna-seq based on a multinomial model. Genome Biology, 20(1), 1–16. 10.1186/s13059-019-1861-6 PMC6927135 31870412

[qlag009-B37] Wagner A., Regev A., & Yosef N. (2016). Revealing the vectors of cellular identity with single-cell genomics. Nature Biotechnology, 34(11), 1145–1160. 10.1038/nbt.3711 PMC5465644 27824854

[qlag009-B38] Warton D. I. (2018). Why you cannot transform your way out of trouble for small counts. Biometrics, 74(1), 362–368. 10.1111/biom.12728 28504821

[qlag009-B39] Weine E., Carbonetto P., & Stephens M. (2024). Accelerated dimensionality reduction of single-cell RNA sequencing data with fastglmpca. Bioinformatics, 40(8), btae494. 10.1093/bioinformatics/btae494 PMC11322042 39110511

[qlag009-B40] Xue J. Y., Zhao Y., Aronowitz J., Mai T. T., Vides A., Qeriqi B., Kim D., Li C., de Stanchina E., Mazutis L., Risso D., & Lito P. (2020). Rapid non-uniform adaptation to conformation-specific kras (g12c) inhibition. Nature, 577(7790), 421–425. 10.1038/s41586-019-1884-x PMC7308074 31915379

[qlag009-B41] Yeung K. Y., & Ruzzo W. L. (2001). Principal component analysis for clustering gene expression data. Bioinformatics, 17(9), 763–774. 10.1093/bioinformatics/17.9.763 11590094

[qlag009-B42] Žurauskienė J., & Yau C. (2016). pcareduce: Hierarchical clustering of single cell transcriptional profiles. BMC Bioinformatics, 17, 1–11. 10.1186/s12859-015-0844-1 PMC4802652 27005807

