# Supplementary material for: Structured factorization for single-cell gene expression data
Source: J R Stat Soc Ser C Appl Stat. 2026 Feb 25;75(5):1133–47. doi: 10.1093/jrsssc/qlag009 (PMC13770536; doi:10.1093/jrsssc/qlag009)
Supplement: Supplementary file 1 [file qlag009_supplementary_data.pdf]

Suplementary Materials for
"Structured factorization for single-cell gene expression data"

Canale, A., Galtarossa, L., Risso, D., Schiavon, L., and Toto, G.

Abstract

Appendix A includes the Gibbs sampling algorithm referenced in Sections 3–4. Appendix B reports additional details and results of the simulation experiments. Appendix C reports additional results related to the CITE scRNA-seq data application. Finally, Appendix D reports the application of COSIN to a dataset related to lung adenocarcinoma scRNA-seq.

An implementation of the Gibbs sampling algorithm is included in the R package `cosin` available at `https://github.com/giovannitoto/cosin`. The R code developed and used in the paper is available at `https://github.com/giovannitoto/cosin_code`.

Appendix A Gibbs sampler algorithms

In this appendix we report the Gibbs sampling algorithm for the COSIN approach referenced in Sections 3–4 of the paper. Detailed settings of the algorithm are also included.

Given k^* the number of factors at iteration t, the algorithm iterates along the following steps

i. Update the elements z_{ij} $(i = 1, \dots, n;\ j = 1 \dots, p)$ sampling independently and in parallel from the truncated normal

$$(z_{ij} \mid -) \sim TN(x_i^T \beta_{jh} + \sum_{h=1}^{k} \eta_{ih}\lambda_{jh}, 1, l_{ij}, u_{ij}),$$

with lower bound $l_{ij} = \log(y_{ij})$ and upper bound $u_{ij} = \log(y_{ij} + 1)$.

ii. Update in parallel the d technical meta-covariate coefficient row vectors $\gamma_{lT}^\top$ $(l = 1, \dots, d)$ sampling from conditionally independent posteriors

$$(\gamma_{lT} \mid -) \sim N_{q_T}\big\{(I_{q_T} + \sigma_\beta^{-2} w_T^\top w_T)^{-1}\sigma_\beta^{-2}(w_T^\top \beta_l),\ (I_{q_T} + \sigma_\beta^{-2} w_T^\top w_T)^{-1}\big\},$$

where $\beta_l^\top$ is the lth p-variate row vector of the coefficient matrix β.

iii. Update in parallel the p covariate coefficient vectors β_j $(j = 1, \dots, p)$ by sampling from the independent full conditional posterior distributions

$$(\beta_j \mid -) \sim N_d\big[(\sigma_\beta^{-2} I_d + x^\top x)^{-1}\{x^\top(z_j - \eta\lambda_j) + \sigma_j^{-2}\Gamma_T w_{jT}\}, (\sigma_\beta^{-2} I_d + x^\top x)^{-1}\big],$$

where η is the $n \times k^*$ matrix of latent scores and z_j is the jth column vector of $z_j = [z_{1j}, \dots, z_{nj}]^\top$. Then, we set $\epsilon = z - x\beta$.

iv. Update, for $i = 1, \dots, n$, the factor η_i according to the posterior full conditional

$$(\eta_i \mid -) \sim N_{k^*}\big\{(I_{k^*} + \Lambda_{k^*}^\top \Sigma^{-1}\Lambda_{k^*})^{-1}\Lambda_{k^*}^\top \Sigma^{-1}\epsilon_i,\ (I_{k^*} + \Lambda_{k^*}^\top \Sigma^{-1}\Lambda_{k^*})^{-1}\big\}.$$

The distribution is conditional to Λ and ϵ, so we can update in parallel the n vectors η_i.

v. Update in parallel the p elements of Σ, by sampling

$$(\sigma_j^{-2} \mid -) \sim \text{Ga}\left\{a_\sigma + \frac{n}{2}, b_\sigma + \frac{1}{2}\sum_{i=1}^{n}(\epsilon_{ij} - \lambda_j^\top \eta_i)^2\right\}.$$

vi. Update the local scale parameters proceeding as follows.

*vi.*i Let $\phi_{jh} = \varphi_{jh}\tilde{\phi}_{jh}$, with φ_{jh} and $\tilde{\phi}_{jh}$ independent a priori and distributed as $\text{Ber}\{\text{logit}^{-1}(w_{jB}^\top \gamma_{hB})\}$ and $\text{Ber}(c_p)$, respectively. Update φ_{jh}, for $j = 1, \dots, p$ and $h = 1, \dots, k^*$, setting $\varphi_{jh} = 1$ if $\phi_{jh} = 1$ and sampling from the full conditional distribution

$$\text{pr}(\varphi_{jh} = u) \propto \begin{cases} 1 - \text{logit}^{-1}(w_{jB}^\top \gamma_{hB}) & \text{for } u = 0, \\ \text{logit}^{-1}(w_{jB}^\top \gamma_{hB})(1 - c_p) & \text{for } u = 1, \end{cases}$$

if $\phi_{jh} = 0$. Given $w_B \Gamma_B$, the elements φ_{jh} $(j = 1, \dots, p,\ h = 1, \dots, k^*)$ are independently distributed and can be updated in parallel.

vi.ii Update each column vector γ_{hB} of Γ_B exploiting the Pólya-Gamma data-augmentation strategy (Polson et al., 2013). Let $\mathrm{pr}(x) \propto \sum_{n=0}^{\infty}(-1)^n A_n (2\pi x^3)^{-0.5}\exp\{-(2n+b)^2(8x)^{-1} - 0.5c^2 x\}$ indicate the probability density function of a Pólya-Gamma distributed random variable $x \sim \mathrm{PG}(b,c)$. For each $h = 1,\ldots,k^*$, generate p independent random variables $d_{j(h)}$ sampling from the full conditional distribution $(d_{j(h)} \mid -) \sim \mathrm{PG}(1, w_{jB}^\top \gamma_{hB}^{(t-1)})$. Let $D_{(h)}$ denote the $p \times p$ diagonal matrix with entries $d_{j(h)}$ $(j = 1,\ldots,p)$. For each $h = 1,\ldots,k^*$, update γ_{hB} sampling from

$$(\gamma_{hB} \mid -) \sim N_{q_B}\{(w^\top D_{(h)} w + I_{q_B})^{-1}(w_B^\top \kappa_h),\, (w_B^\top D_{(h)} w + I_{q_B})^{-1}\},$$

where κ_h is a p-dimensional vector with the j-th entry equal to $\varphi_{jh} - 0.5$. Given $\varphi_{jh}\,(j = 1,\ldots,p;\ h = 1,\ldots,k^*)$, we can update in parallel all the vectors $\gamma_{hB}\,(h = 1,\ldots,k^*)$.

vii. Let $\tilde\lambda_{jh}$ denote the continuous underlying loadings element such that $\lambda_{jh} = \phi_{jh}\rho_h\tilde\lambda_{jh}$ $(h = 1,\ldots,k^*)$. Then, update the elements $\tilde\lambda_{jh}$ by sampling from the independent full conditional posterior distributions of the row vector $\tilde\lambda_j$ $(j = 1,\ldots,p)$,

$$(\tilde\lambda_j \mid -) \sim \mathcal{N}_{k^*}\big\{(D_j^{-1} + F_j\eta^\top\eta F_j)^{-1}F_j\eta^\top\epsilon_j,\, (D_j^{-1} + F_j\eta^\top\eta F_j)^{-1}\big\},$$

where $D_j^{-1} = \mathrm{diag}(\vartheta_1^{-1},\ldots,\vartheta_{k^*}^{-1})$, $F_j = \mathrm{diag}(\rho_1\phi_{1j},\ldots,\rho_{k^*}\phi_{k^*})$, and ϵ_j is the column vector $\epsilon = [\epsilon_{1j},\ldots,\epsilon_{nj}]^\top$. The distribution is conditional to factor scores η and to the scale matrix and vector Φ and θ, thus we can update in parallel the p vectors $\tilde\lambda_j$. Finally, set $\lambda_{jh} = \phi_{jh}\rho_h\tilde\lambda_{jh}$ for any $j = 1,\ldots,p$ and $h = 1,\ldots,k^*$.

viii. Update the factor-specific scale parameters proceeding as follows.

viii..I Following Legramanti et al. (2020), define the independent indicators ξ_h $(h = 1,\ldots,p)$ with prior $\mathrm{pr}(\xi_h = l) = u_l$. Update the augmented data ξ_h by sampling from the full conditional distribution

$$\mathrm{pr}(\xi_h = l) \propto \begin{cases} u_l\,\mathrm{pr}_N\{\mathrm{vec}(\epsilon); \mathrm{vec}(\eta\Lambda) - \mathrm{vec}(\eta_h\lambda_h^\top), I_{np}\} & \text{for} \quad l = 1,\ldots,h \\ u_l\,\mathrm{pr}_N\{\mathrm{vec}(\epsilon); \mathrm{vec}(\eta\Lambda) + (1-\rho_h^{(t-1)})\mathrm{vec}(\eta_h\lambda_h^{*\top}), I_{np}\} & \text{for} \quad l = h+1,\ldots,k^*, \end{cases}$$

where we define the row vector $\lambda_h^{*\top}$ such that $\lambda_h^\top = \rho_h\lambda_h^{*\top}$. Then, $\rho_h = 1$ if $\xi_h > h$, else $\rho_h = 0$. The full conditional distribution of ξ_h depends on the value of $\rho_l\,(l = 1,\ldots,k^*)$, implying to immediately set $\lambda_h^\top = \rho_h\lambda_h^{*\top}$ and to update ρ_h sequentially with respect to the index $h = 1,\ldots,k^*$.

viii..II Update the parameter ϑ_h $(h = 1,\ldots,k^*)$ by sampling ϑ_h^{-1} from the full conditional distribution $\mathrm{Ga}(a_\theta + 0.5p, b_\theta + 0.5\sum_{j=1}^{p}\tilde\lambda_{jh}^2)$.

viii..III For $l = 1,\ldots,k^*-1$, sample v_l from

$$(v_l \mid -) \sim \mathrm{Be}\big\{1 + \sum_{h=1}^{k^*}\mathbb{1}(\xi_h = l), \alpha + \mathbb{1}(\xi_h > l)\big\},$$

while set $v_{k^*} = 1$. Since, given u, the distribution are conditionally independents, we can update the k^* elements of the vector v in parallel. Finally, update $u_l = v_l\prod_{m=1}^{l-1}(1-v_m)$, for $l = 1,\ldots,k^*$.

ix. Update the local scale by sampling from the full conditional distributions of ϕ_{jh}. If $h \in \{1, \ldots, k^* : \rho_h = 0\}$, then sample from the Bernoulli defined as

$$\mathrm{pr}(\phi_{jh} = \xi) = \begin{cases} 1 - \mathrm{logit}^{-1}(w_{jB}^\top \gamma_{hB})\, c_p & \text{for } \xi = 0 \\ \mathrm{logit}^{-1}(w_{jB}^\top \gamma_{hB})\, c_p & \text{for } \xi = 1. \end{cases}$$

Given the linear predictor matrix $w_B \Gamma_B$, we can update in parallel ϕ_{jh} for $j = 1, \ldots, p$ and $h \in \{1, \ldots, k^* : \rho_h = 0\}$. If $h \notin \{1, \ldots, k^* : \rho_h = 0\}$, sample from

$$\mathrm{pr}(\phi_{jh} = \xi) \propto \begin{cases} \{1 - \mathrm{logit}^{-1}(w_{jB}^\top \gamma_{hB})\, c_p\}\, \mathrm{pr}_N \left(\epsilon_j; \eta\lambda_j - \phi_{jh}^{(t-1)} \tilde{\lambda}_{jh}\eta_h, I_n\right) & \text{for } \xi = 0 \\ \mathrm{logit}^{-1}(w_{jB}^\top \gamma_{hB})\, c_p\, \mathrm{pr}_N \left\{\epsilon_j; \eta\lambda_j + \left(1 - \phi_{jh}^{(t-1)}\right) \tilde{\lambda}_{jh}\eta_h, I_n\right\} & \text{for } \xi = 1. \end{cases}$$

where $\mathrm{pr}_N(x; \mu, I_n)$ is the multivariate density function of the n-variate Gaussian distribution with mean μ, variance equal to the identiy matrix, and evaluated at x. We use $\phi_{jh}^{(t-1)}$ to denote the parameter ϕ_{jh} sampled at the previous iteration of the Gibbs and η_h indicating the hth factor score vector. The distribution of each ϕ_{jh} depends on $w_B \Gamma_B$ and on the elements ϕ_{lj} $(l = 1, \ldots, h)$ via λ_j. Therefore, the update is sequential with respect to the index h and requires to set $\lambda_{jh} = \phi_{jh}\tilde{\lambda}_{jh}$ after having sampled ϕ_{jh} for any $h \notin \{1, \ldots, k^* : \rho_h = 0\}$. On the other hand, we can update in parallel with respect to the index $j = 1, \ldots, p$.

The results reported in the lung adenocarcinoma data application and in simulation studies are obtained running the algorithms for 20000 iterations, while discarding the first 5000 iterations. Then, we thin the Markov chain, saving every 2-th sample. We adapt the number of active factors at iteration t with probability $\mathrm{pr}(t) = \exp(-1 - 5\, 10^{-4} t)$ after at least 100 iterations. In simulation studies, we consider a single vector w_B of biological meta-covariates as described in the main paper.

Appendix B Simulation details

We report the values of the hyperparameters for the simulation of Section 3. We set $\alpha = 5$, $\sigma_\gamma^2 = 1$, $a_\theta = b_\theta = 1$, and $c_p = 0.5$. This is equivalent to set the number of expected factors to $\alpha = 5$ and to set unitary prior expectation for the variance of both the coefficients γ and the factor loadings λ_{jh}. The value $c_p = 0.5$ promotes sparsity within columns of Λ at 50%. The results are not sensitive to mild variations of the above values.

The estimated contributions of each model are ordered to minimise the sum of contribution RMSEs, while the number of latent components k in GLM-PCA models is set to the value that minimises the MAE.

Additionally, we report: the performances of the competing models in clustering scenarios under correct specification of the number of latent dimensions (Figure A1); the full table referenced in Section 3 of the paper reporting the predictive performances of the two competing models (Table A1).

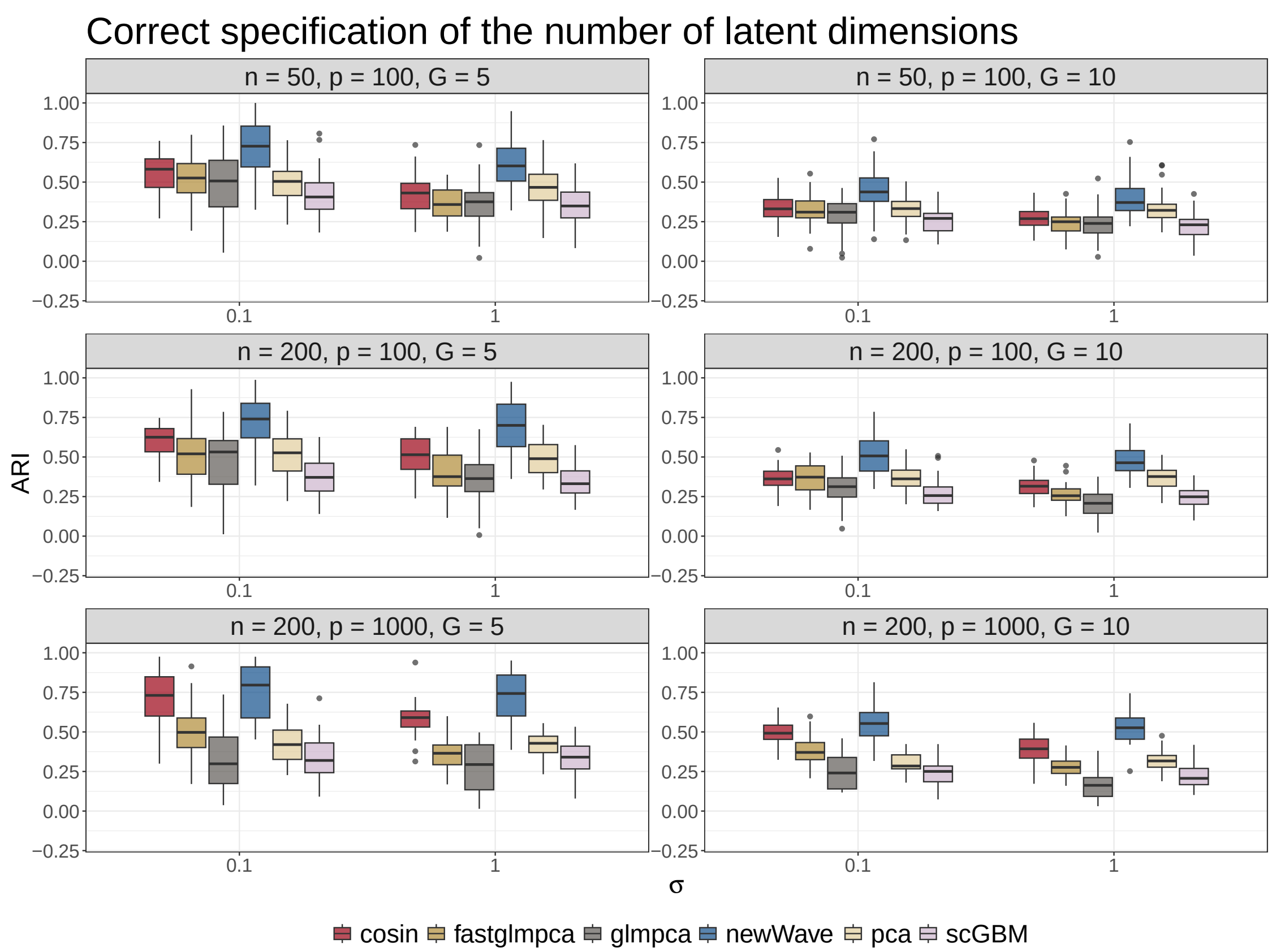

Figure A1 : Boxplots of ARI of the competing models under different values of (n, p), σ^2 and G. Clustering is obtained applying a G-means clustering to the first latent dimensions estimated by each method. The number of latent components is set equal to the number of components k used to generate the data.

Table A1: Median of MAE and of the estimated number of active factors k^* in 50 replicates, with varying (n, p, σ). Interquartile range is reported in parenthesis.

(n, p, σ)	COSIN w/o metacov.	COSIN metacov.	GLM-PCA* w/o metacov.
MAE			
$(50, 100, 0.10)$	0.7464	0.7544	1.4730
	(0.5243)	(0.5076)	(1.4275)
$(50, 100, 1.00)$	4.6107	4.5624	6.1408
	(2.8316)	(2.8434)	(2.8640)
$(200, 100, 0.10)$	0.5055	0.5071	1.3518
	(0.2754)	(0.2776)	(1.1313)
$(200, 100, 1.00)$	4.5456	4.5388	7.3334
	(2.6220)	(2.6028)	(4.1877)
$(200, 1000, 0.10)$	0.4196	0.4170	1.4331
	(0.1119)	(0.1197)	(0.7778)
$(200, 1000, 1.00)$	4.7887	4.7921	6.9329
	(1.4916)	(1.5049)	(2.4602)
k^*			
$(50, 100, 0.10)$	3.0000	3.0720	8.0000
	(1.0000)	(1.0000)	(1.0000)
$(50, 100, 1.00)$	3.0000	3.0000	3.0000
	(0.0000)	(0.0000)	(2.0000)
$(200, 100, 0.10)$	3.0000	3.0000	8.0000
	(0.0654)	(0.0039)	(0.7500)
$(200, 100, 1.00)$	3.0000	3.0000	2.0000
	(0.0000)	(0.0000)	(1.7500)
$(200, 1000, 0.10)$	10.2548	10.6424	8.0000
	(1.0000)	(1.3267)	(0.0000)
$(200, 1000, 1.00)$	4.0000	5.0000	4.0000
	(0.2030)	(0.0000)	(2.0000)

* Since the R package `glmpca` does not allow for out-of-sample imputation, we adopt the GLM-PCA approximation proposed by Townes et al. (2019), i.e. we perform principal component analysis on Pearson residuals of the null Poisson model.

Appendix C CITE-seq application: additional plots and tables

This appendix includes additional tables and figures for CITE-seq data application referenced in Section 4 of the paper.

Table A2: List of biological pathways considered as biological meta-covariates.

Immune pathway	Number of genes
Chemokine signaling pathway	154
Complement and coagulation cascades	31
Platelet activation	108
Antigen processing and presentation	39
Neutrophil extracellular trap formation	91
Toll like receptor signaling pathway	77
NOD like receptor signaling pathway	132
RIG I like receptor signaling pathway	51
Cytosolic DNA sensing pathway	28
C type lectin receptor signaling pathway	57
Natural killer cell mediated cytotoxicity	90
IL 17 signaling pathway	70
Th1 and Th2 cell differentiation	77
Th17 cell differentiation	85
T cell receptor signaling pathway s	95
B cell receptor signaling pathway	75
Fc epsilon RI signaling pathway	53
Fc gamma R mediated phagocytosi	90
Leukocyte transendothelial migration	70
Intestinal immune network for IgA production	23

Largest pathway	Number of genes
Metabolic pathways	1119
Herpes simplex virus 1 infection	420
Pathways of neurodegeneration multiple diseases	335
Pathways in cancer	394

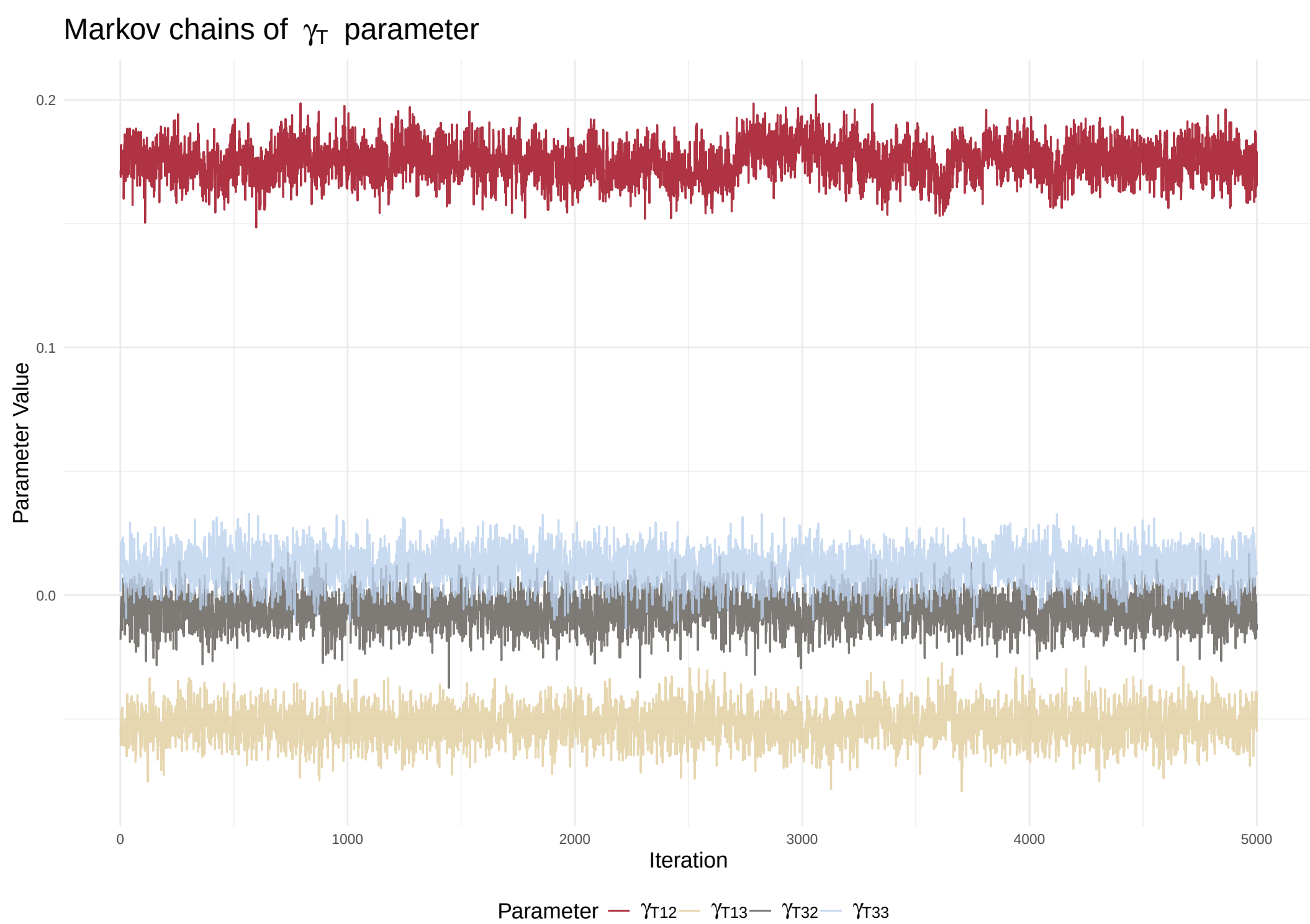

Figure A2 : Markov chains evolution of technical meta-covariate coefficient parameters Γ_T.

Table A3: Summary results of the posterior mean of β matrix and number of genes characterised by 0.9-level credible intervals not including zero.

Covariate	Min.	$Q_0.25$	$Q_0.5$	$Q_0.75$	Max.	0.9-level genes
Genes-specific intercept	−4.121	−2.173	−1.702	−1.104	3.383	2580
Number of genes	−0.408	0.113	0.178	0.234	0.707	1775
Total count per cell	−0.613	−0.121	−0.053	0.017	0.809	881

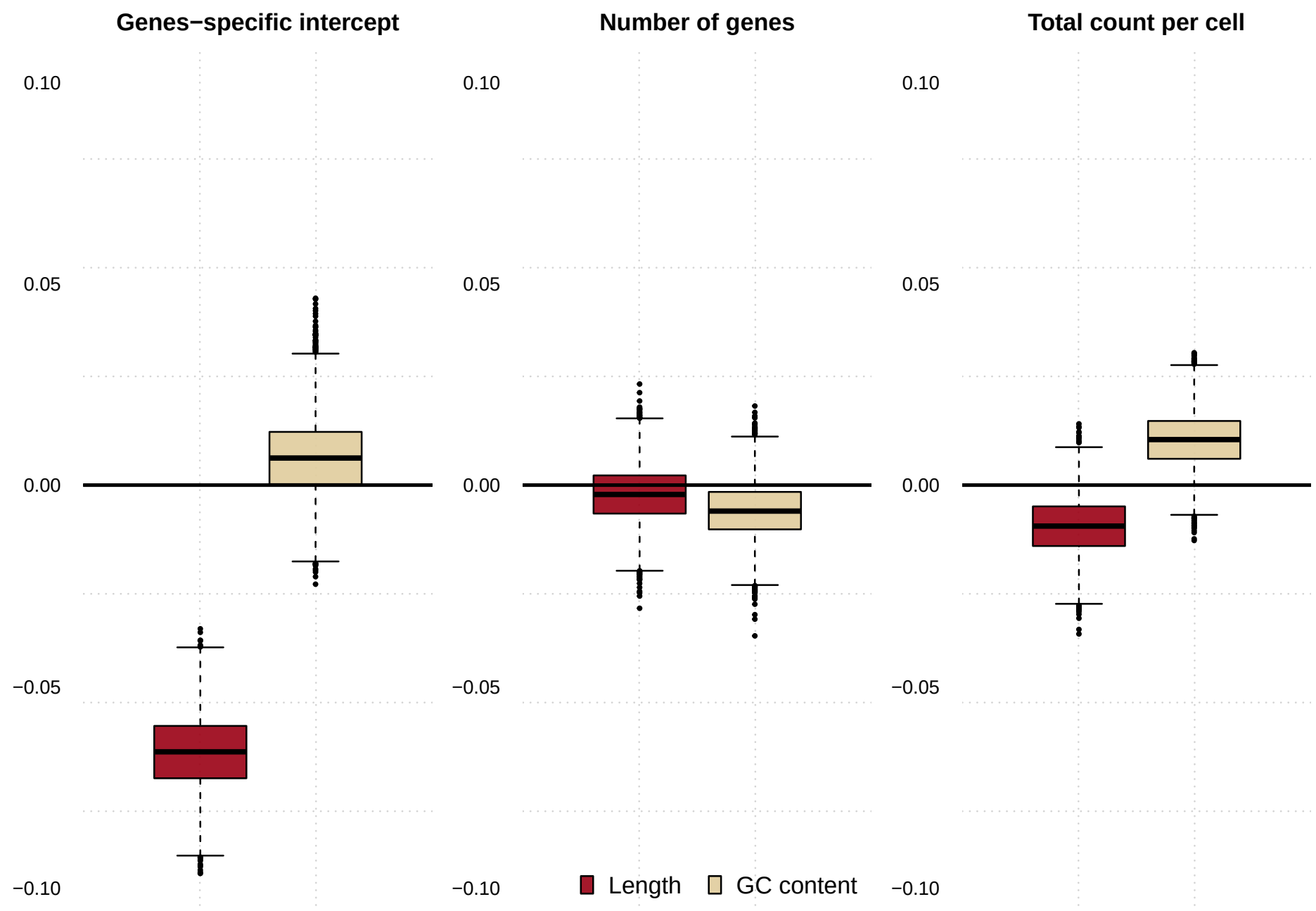

Figure A3 : Posterior distributions of the parameter Γ_T entries. Red and yellow boxplots indicate the influence of the genes length and the genes GC content, respectively, on the covariate effects.

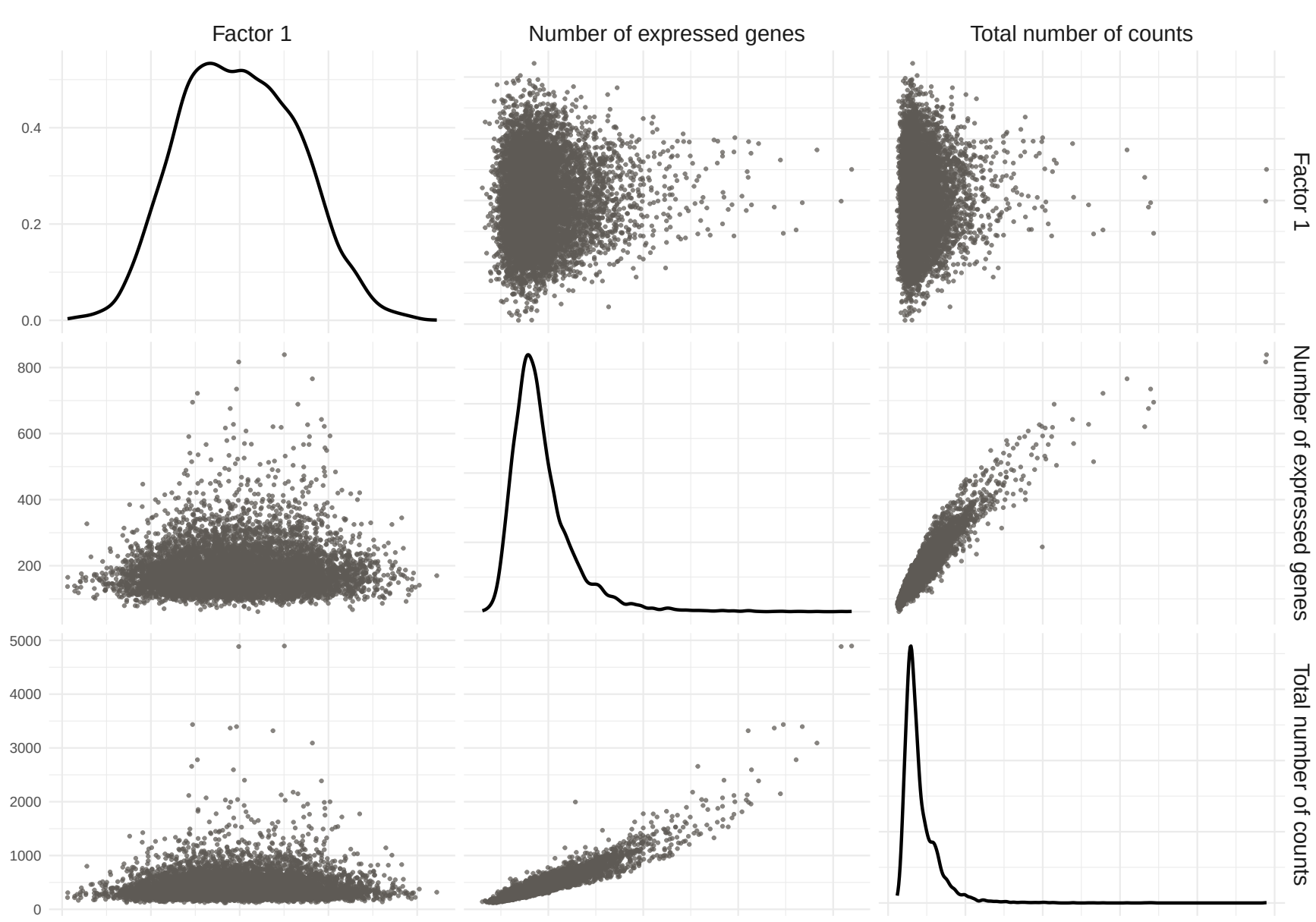

Figure A4 : Density and pair plots of covariates (four panels in the bottom right) and the scores of the first latent factor from a representative posterior draw (first row and column).

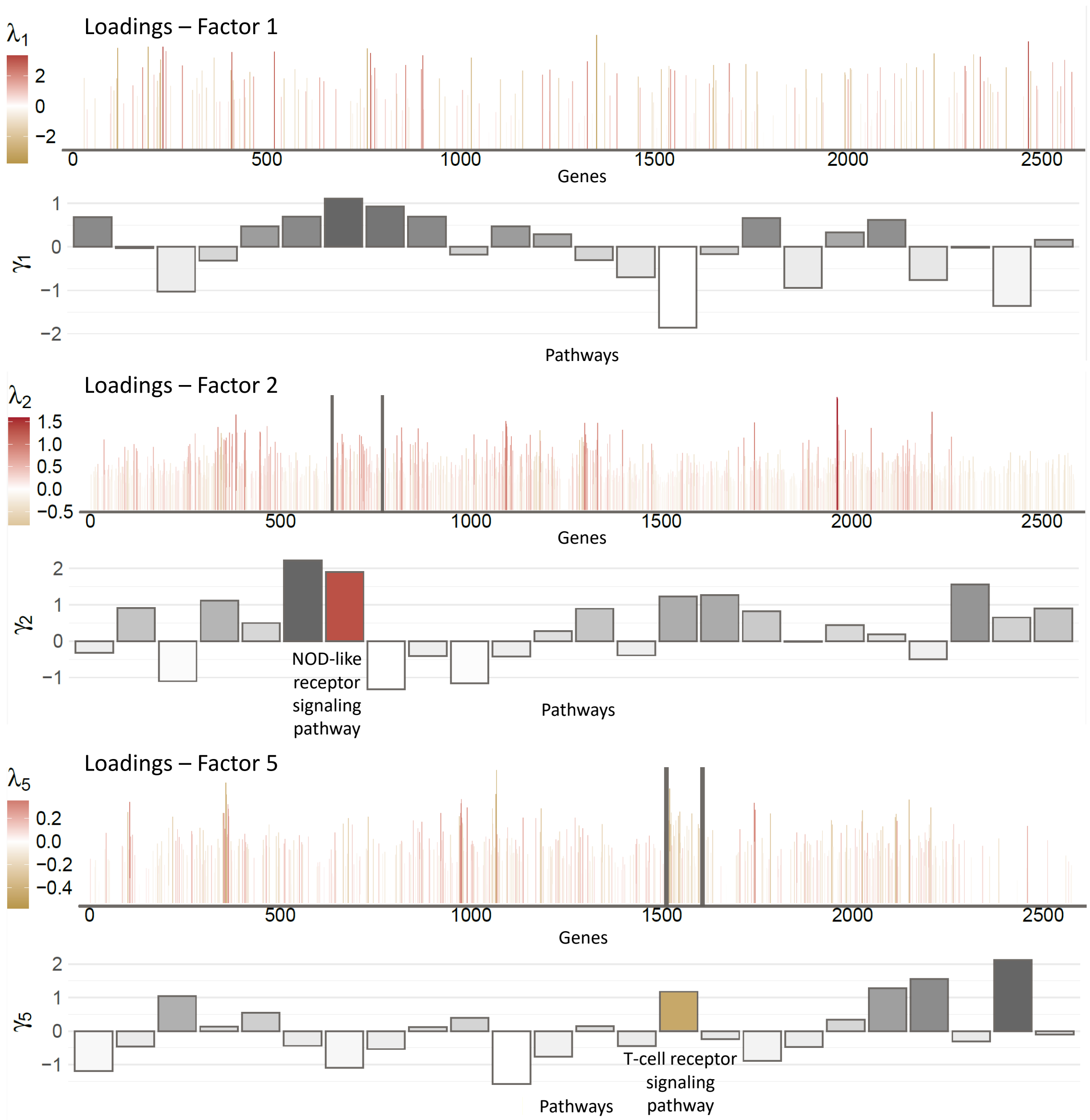

Figure A5 : Barplots of the loadings and biological meta-covariate coefficients γ_B of first, second, and fifth factors of a representative posterior draw. The grey vertical lines on the loading barplots delimit the genes associated to the pathways highlighted in the corresponding γ barplots.

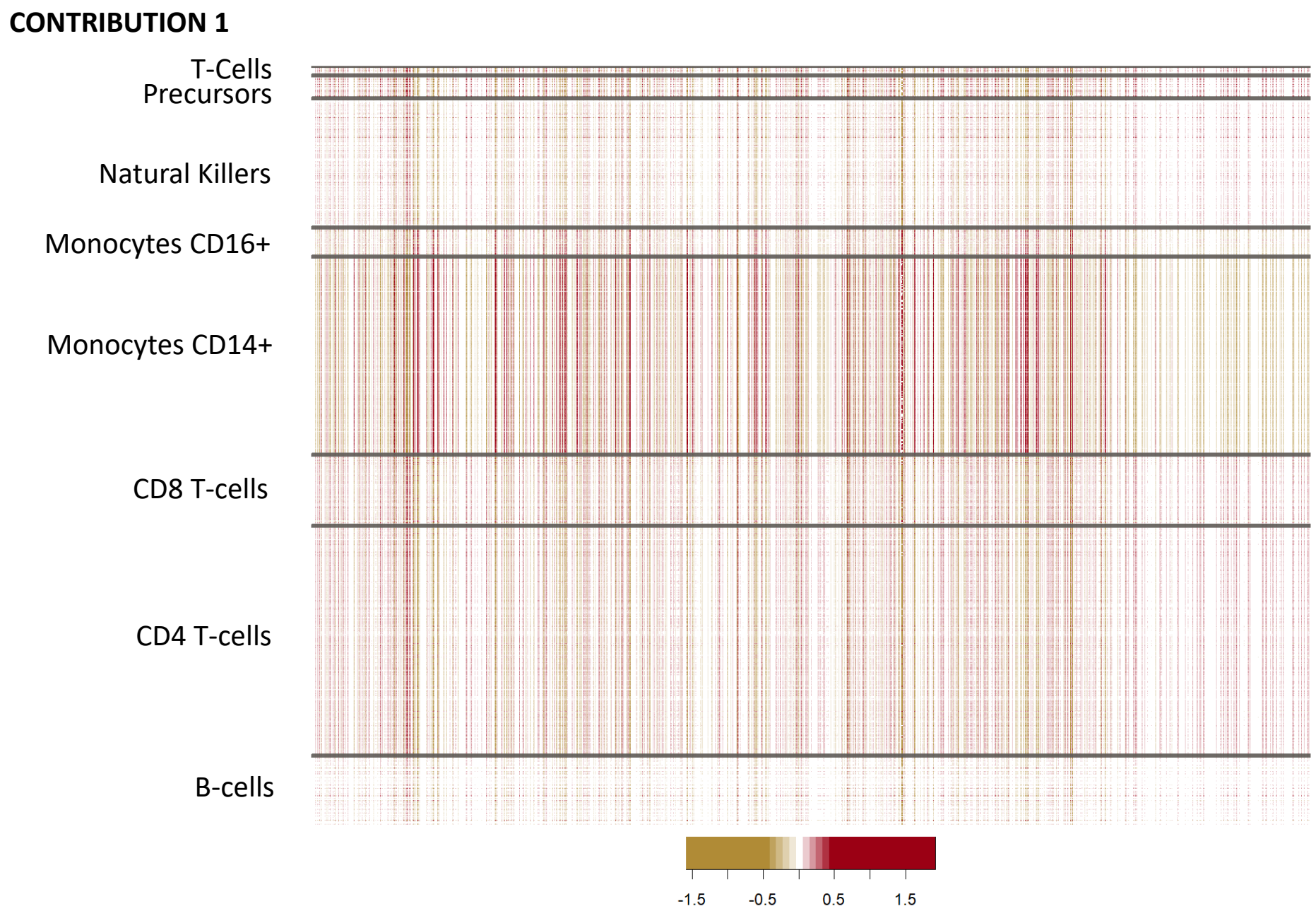

Figure A6 : First rank-one contribution matrix. Rows are re-arranged according to cell type.

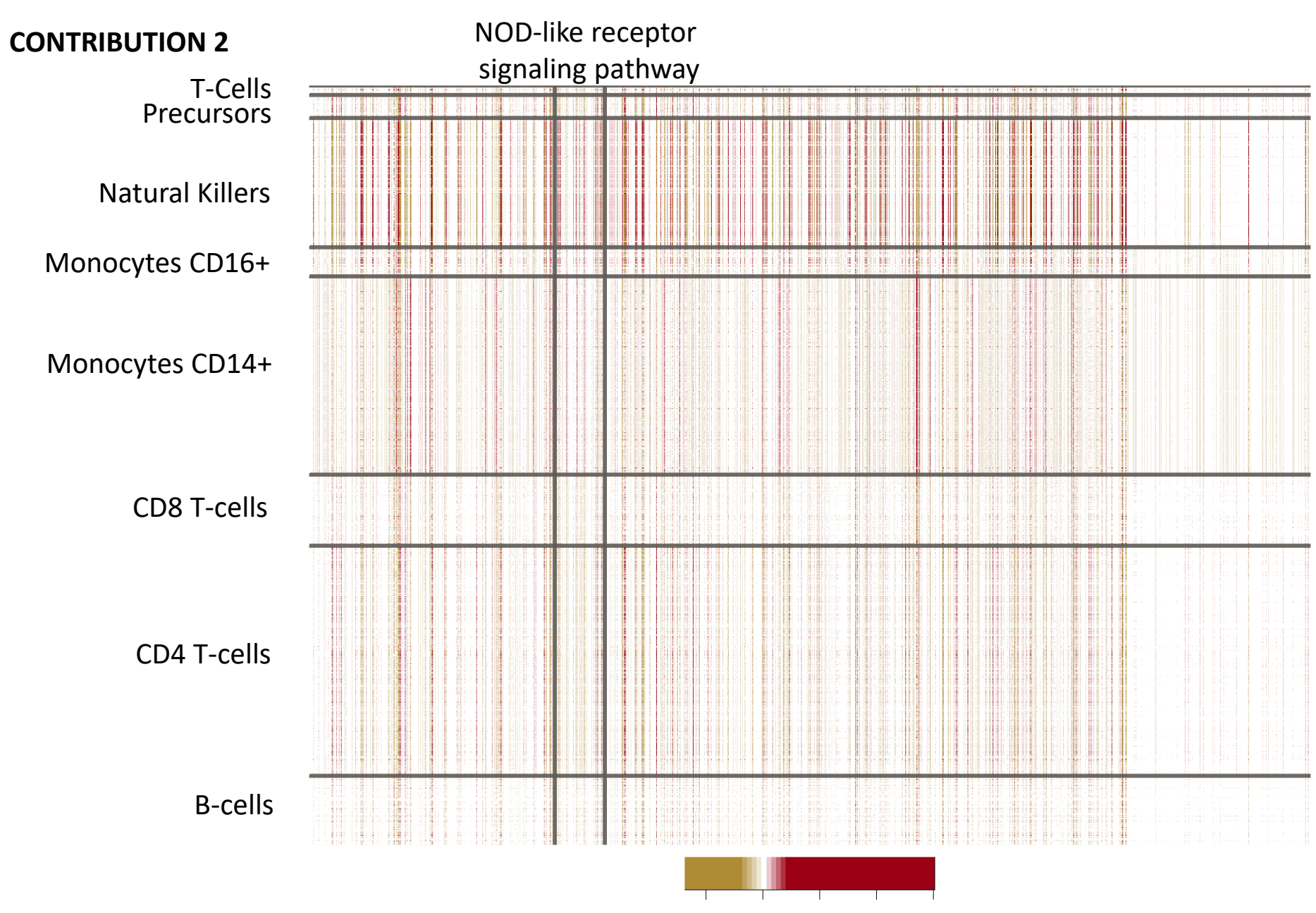

Figure A7 : Second rank-one contribution matrix. Rows are re-arranged according to cell type. Genes are ordered according to the NOD-like receptor signaling pathway, with genes belonging to this pathway separated by grey vertical lines.

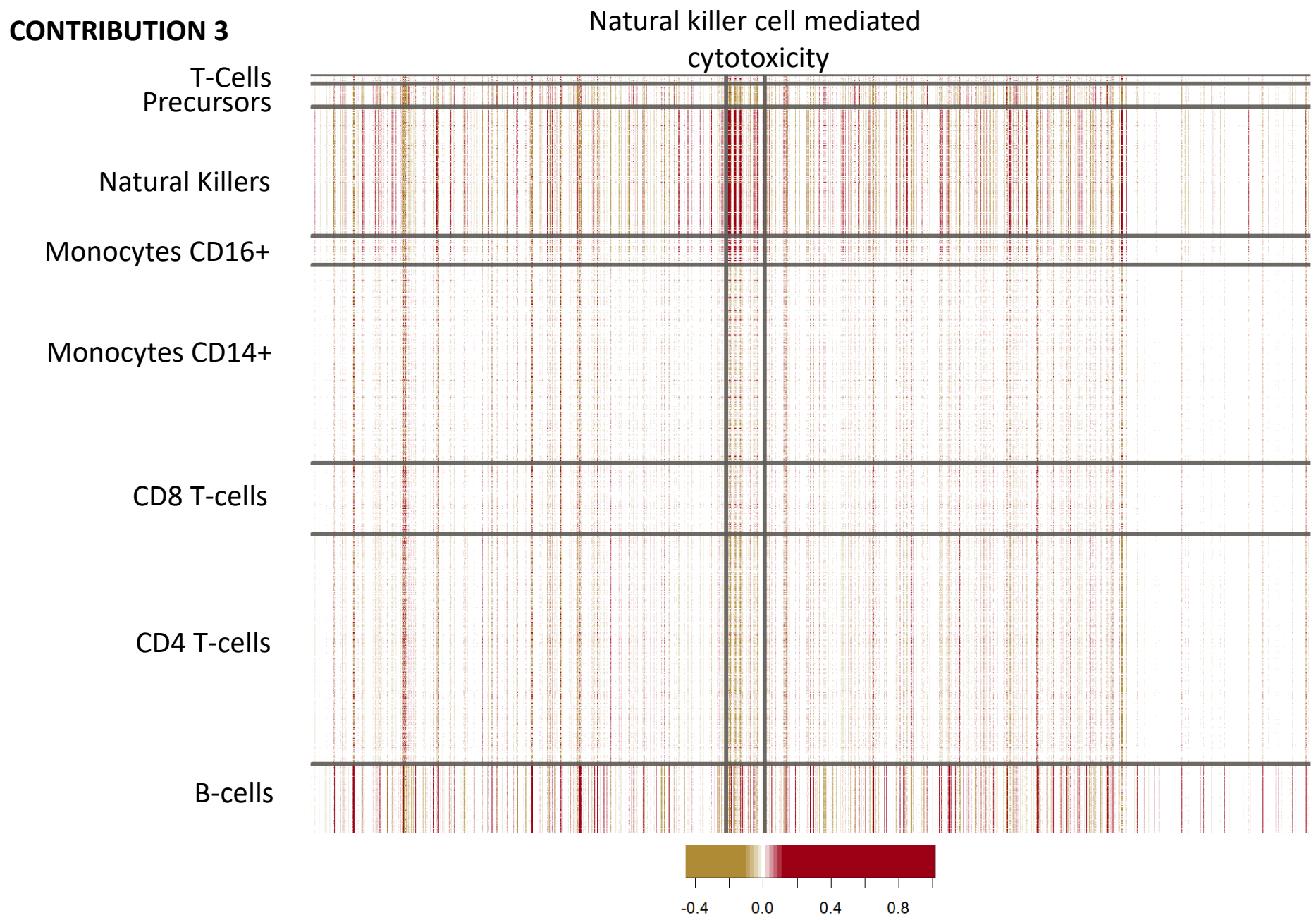

Figure A8 : Third rank-one contribution matrix. Rows are re-arranged according to cell type. Genes are ordered according to the pathway of natural killer cell mediated cytotoxicity, with genes belonging to this pathway separated by grey vertical lines.

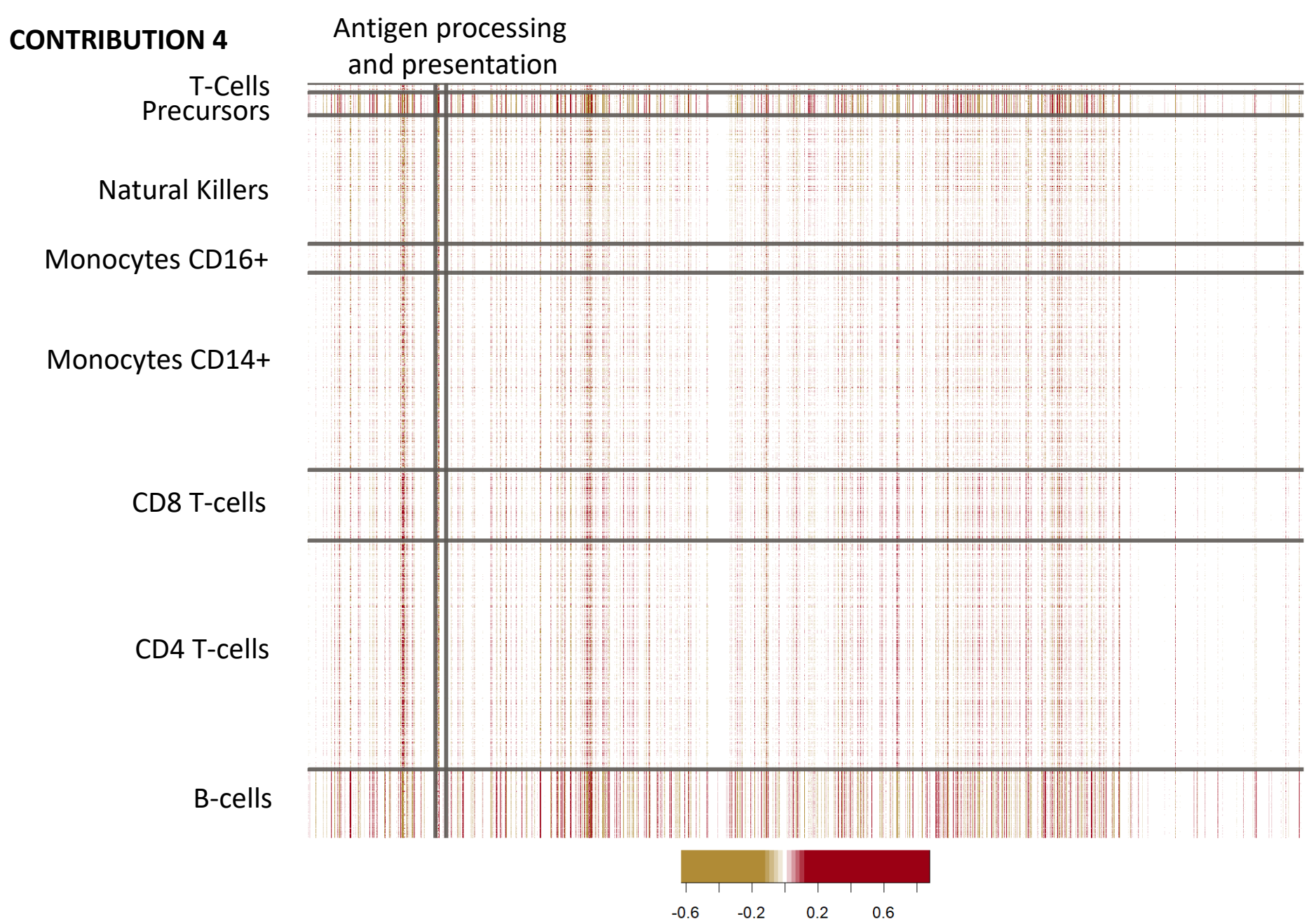

Figure A9 : Fourth rank-one contribution matrix. Rows are re-arranged according to cell type. Genes are ordered according to the Antigen processing and presentation pathway, with genes belonging to this pathway separated by grey vertical lines.

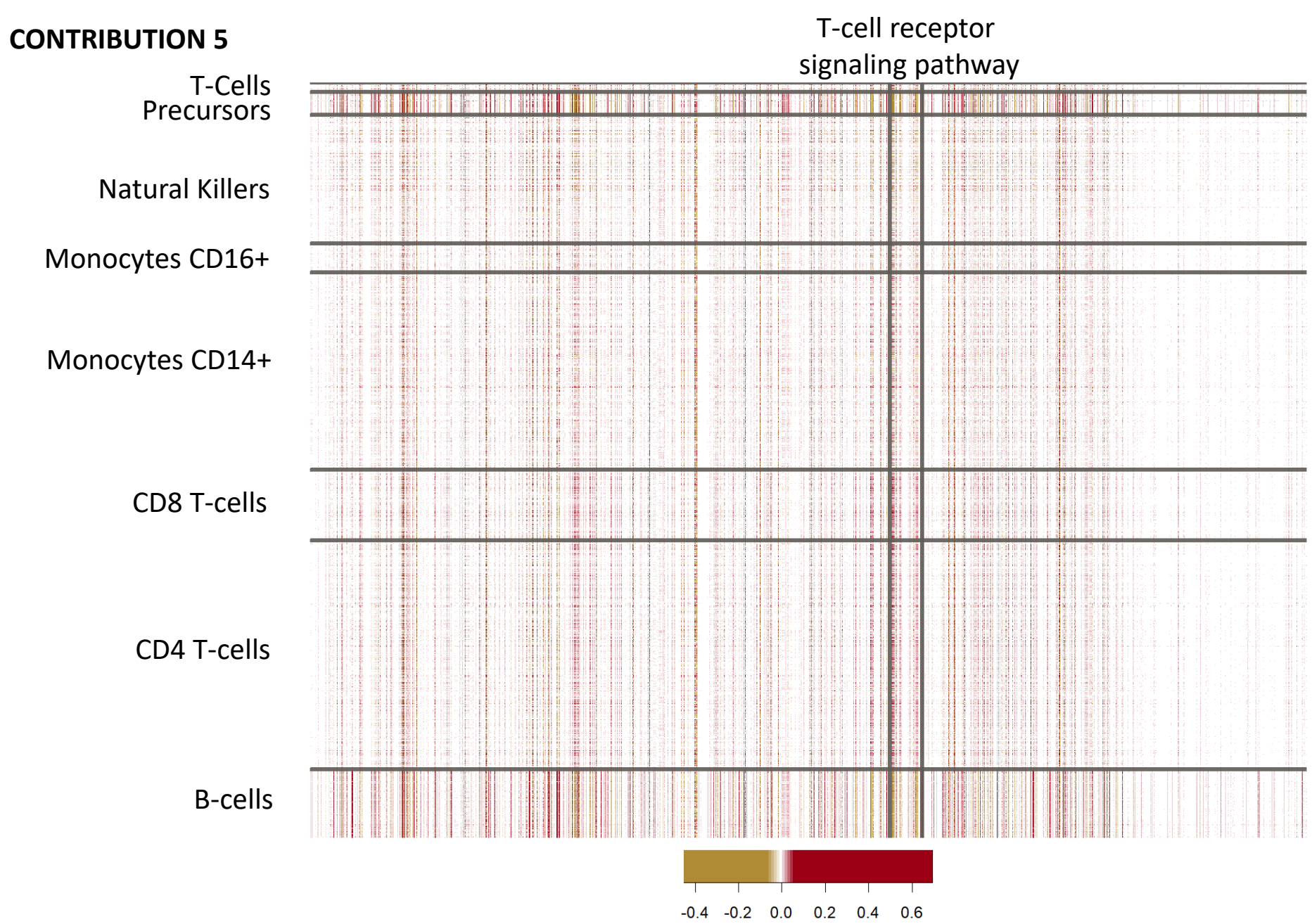

Figure A10 : Fifth rank-one contribution matrix. Rows are re-arranged according to cell type. Genes are ordered according to the T-cell receptor signaling pathway, with genes belonging to this pathway separated by grey vertical lines.

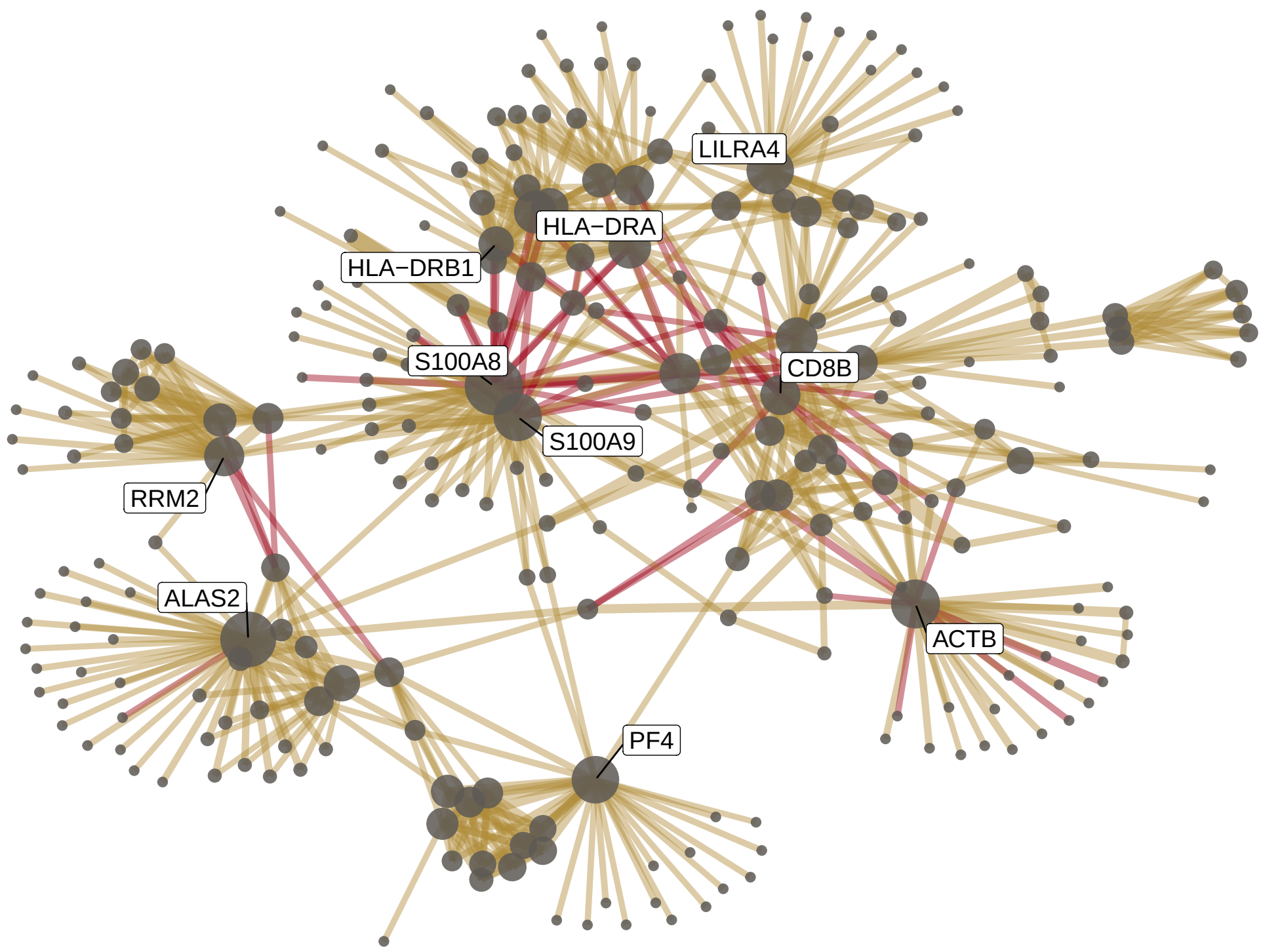

Figure A11 : Graphical representation based on the posterior mean of the inverse of the correlation matrix estimated by the model. Edges are proportionally thick according to the absolute partial correlations between genes and are coloured based on the sign of the partial correlations. Values below 0.025 are not reported. Nodes are positioned using a Fruchterman–Reingold force-directed algorithm.

Appendix D Lung adenocarcinoma application

We analyse a subset of the study of three lung adenocarcinoma cell lines measured by scRNA-seq by Tian et al. (2019). The original data are high quality in terms of exon mapping and unique transcript counts per cell and have already undergone pre-processing and quality control by the original authors using the `scPipe` workflow (Tian et al., 2018). Cells with high percentages of mitochondrial genes and less than 1000 detected genes are excluded and only genes belonging to the 20 largest pathways are retained. The resulting data consist of a gene expression matrix of $n = 199$ cells and $p = 949$ genes. Along with this gene expression matrix, quality control information about the cells x and the genes w_T are also available. The matrix x includes technical features, such as the number of unaligned reads, the number of reads mapped to exons or introns, the number of expressed genes, and the total number of counts, leading to $d = 9$ covariates. The full list of covariates is reported in Table A5. In addition, cell line information is also available, indicating for any cell if it belongs to cancer lines H1975, H2228, or HCC827. We chose not to use such information as a covariate in the model, to mimic a typical situation in scRNA-seq, in which the identity of the cells is not known in advance. Hence, it represents a useful benchmark to assess the capacity of the model to reconstruct unobserved covariates in high-dimensional settings. Technical meta-covariate matrix w_T includes the length and the GC-content of each gene. Consistently with the motivations previously mentioned, for each gene j, we also define a biological meta-covariate binary vector w_{jB} with m entry equal to 1 if the gene j belongs to the m biological pathway. The list of $q_B = 20$ pathways considered is reported in Table A4.

Table A4: List of biological pathways considered as meta-covariates

Pathway	Number of genes
Metabolic pathways	313
Pathways of neurodegeneration multiple diseases	153
Amyotrophic lateral sclerosis	121
Alzheimer disease	116
Pathways in cancer	109
Parkinson disease	108
COVID 19 pathway	103
Salmonella infection	100
Shigellosis	92
PI3K-AKT signaling pathway	79
Human papillomavirus infection	77
Huntington disease	73
Regulation of actin cytoskeleton	71
Cell cycle	69
Focal adhesion	65
Tight junction	64
MAPK signaling pathway	63
Human cytomegalovirus infection	63
Human immunodeficiency virus 1 infection	62
Proteoglycans in cancer	62

We apply COSIN with latent continuous Gaussian variable z specified as in Section 2 of the main paper. Given the high dimensionality of the data set, we may expect a sufficiently large number of latent factors, hence we set $\alpha = 10$. To favour variable selection, we shrink covariate coefficients setting $\sigma_\beta^2 = 1/3$. Further hyper-parameters are set consistently with the simulation study.

Table A5: Summary results of the posterior mean of β matrix and number of genes characterised by 0.9-level credible intervals not including zero.

Covariate	Min.	$Q_0.25$	$Q_0.5$	$Q_0.75$	Max.	0.9-level genes
Intercept	−0.504	0.089	0.164	0.279	0.579	85
Unaligned	−0.371	−0.062	0.004	0.067	0.326	0
Aligned unmapped	−0.627	−0.067	0.01	0.071	0.413	14
Mapped to exon	−0.218	−0.013	0.037	0.088	0.338	0
Mapped to intron	−0.296	−0.075	−0.009	0.055	0.378	22
Ambiguous mapping	−0.379	−0.051	0.018	0.086	0.369	0
Mapped to MT	−0.231	−0.045	−0.001	0.044	0.292	20
Number of genes	−0.338	0.050	0.117	0.189	0.517	12
Total count	−0.162	0.012	0.051	0.093	0.261	0

First, we discuss the results obtained for the mean of the process and the cell covariate effects. Summaries of the matrix β are reported in Table 5. The last column illustrates that only a small proportion of β's for any covariate has 0.9-level posterior credible intervals not including zero. This fact suggests a low importance of the control quality cell features, confirming that the data are of high quality. As one may expect, the total number of genes detected per cell seems positively associated to the gene expression, but with just few genes where such effect is relevant. The variables describing the number of reads mapped to introns and to mitochondrial genes are those relevantly impacting the highest number of genes. The direction of such impacts varies on different genes, with variability partially explained by the technical characteristics of the genes, i.e. the length and the GC-content of the genes.

Figure A11 displays the posterior distributions of the Γ_T coefficients, illustrating the influence of meta-covariates on the covariate effects. The GC-content and the length of the genes seem to have an impact mainly on the effect of the number of reads mapped to introns and those with ambiguous mapping.

Additional insights can be gained by exploring the structure of the sparse covariance matrix $\text{var}(z_j) = \Lambda\Lambda^\top + \Sigma$. The graph constructed from the posterior mean of its inverse (i.e. the partial correlation matrix), reported in Figure A12, reveals the presence of gene communities with genes belonging to the same pathway having the tendency of being clustered together. For instance, the cluster observed at the bottom of the graph is mainly constituted by the genes belonging to the cell cycle and metabolic and cancer pathways, while the genes related to the COVID-19 pathway are mainly distributed in the communities at the top right corner of the graph. This structure is favoured, yet not imposed, by the dependence on the meta-covariates induced by the structured increasing shrinkage specification on the loadings matrix Λ. In addition, the graph highlights the genes which stand out as hub nodes, since are correlated with large groups of genes, or link different clusters. Notable hub genes include cell cycle kinases, such as CDK1 and CDK4, essential for G1/S and G2/M phase transitions, and for cell cycle control (Nurse et al., 1998). Because of the central role of the cell cycle in cancer progression, these genes have been proposed as therapeutic targets for

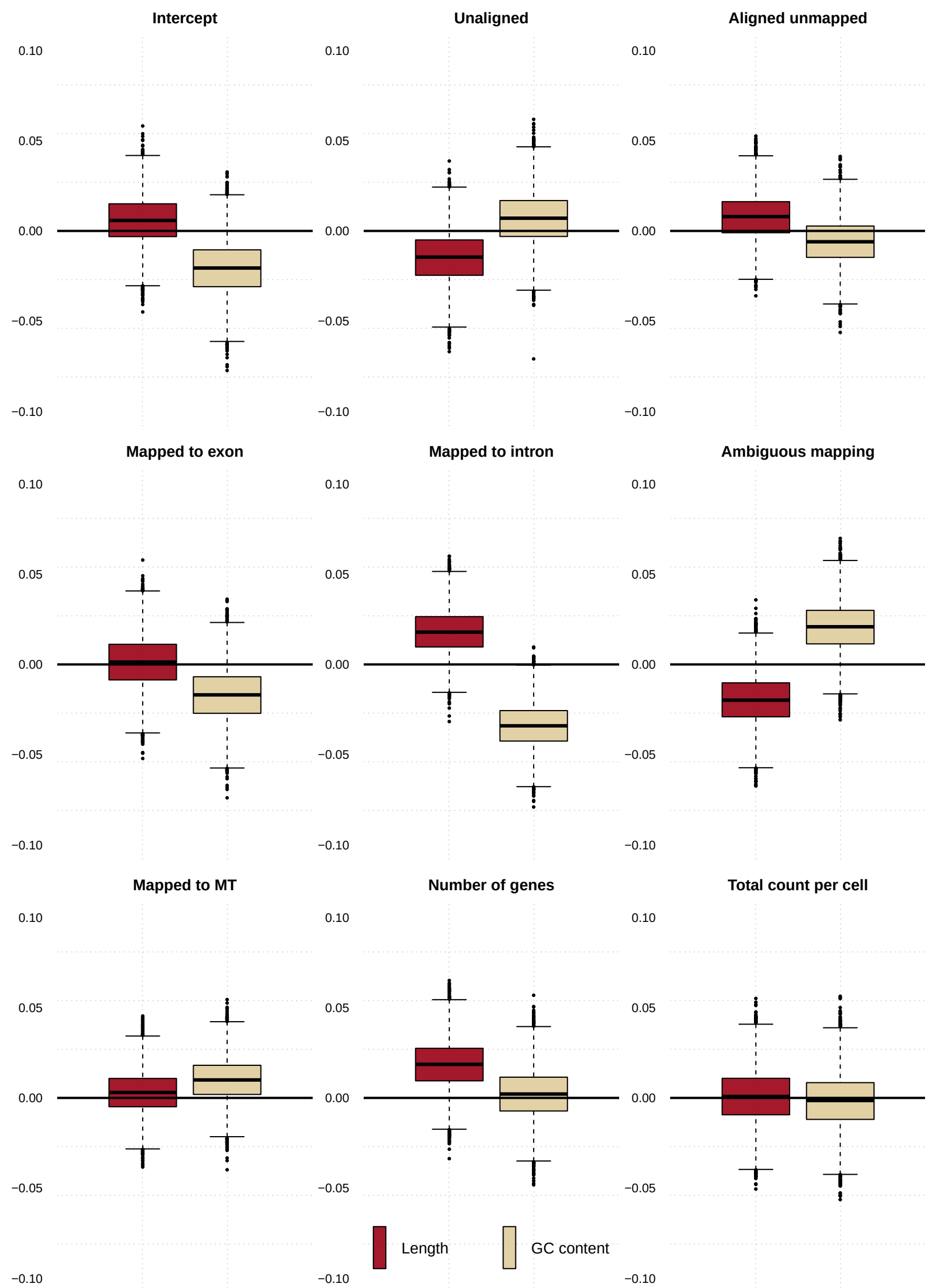

Figure A12 : Posterior distributions of the parameter Γ_T entries. Red and yellow boxplots indicate the influence of the genes length and the genes GC content, respectively, on the covariate effects.

inhibitors in lung adenocarcinoma treatment (Asghar et al., 2015). Other hub nodes that have been associated with cancer in the literature include HSP90AA1 and ITGB1, which belong to pathways in cancers and to the PI3K-AKT signaling pathway, and B2M that is involved in immune resistance promoting cancer transformation of the cells and escape from therapies (Pereira et al., 2017).

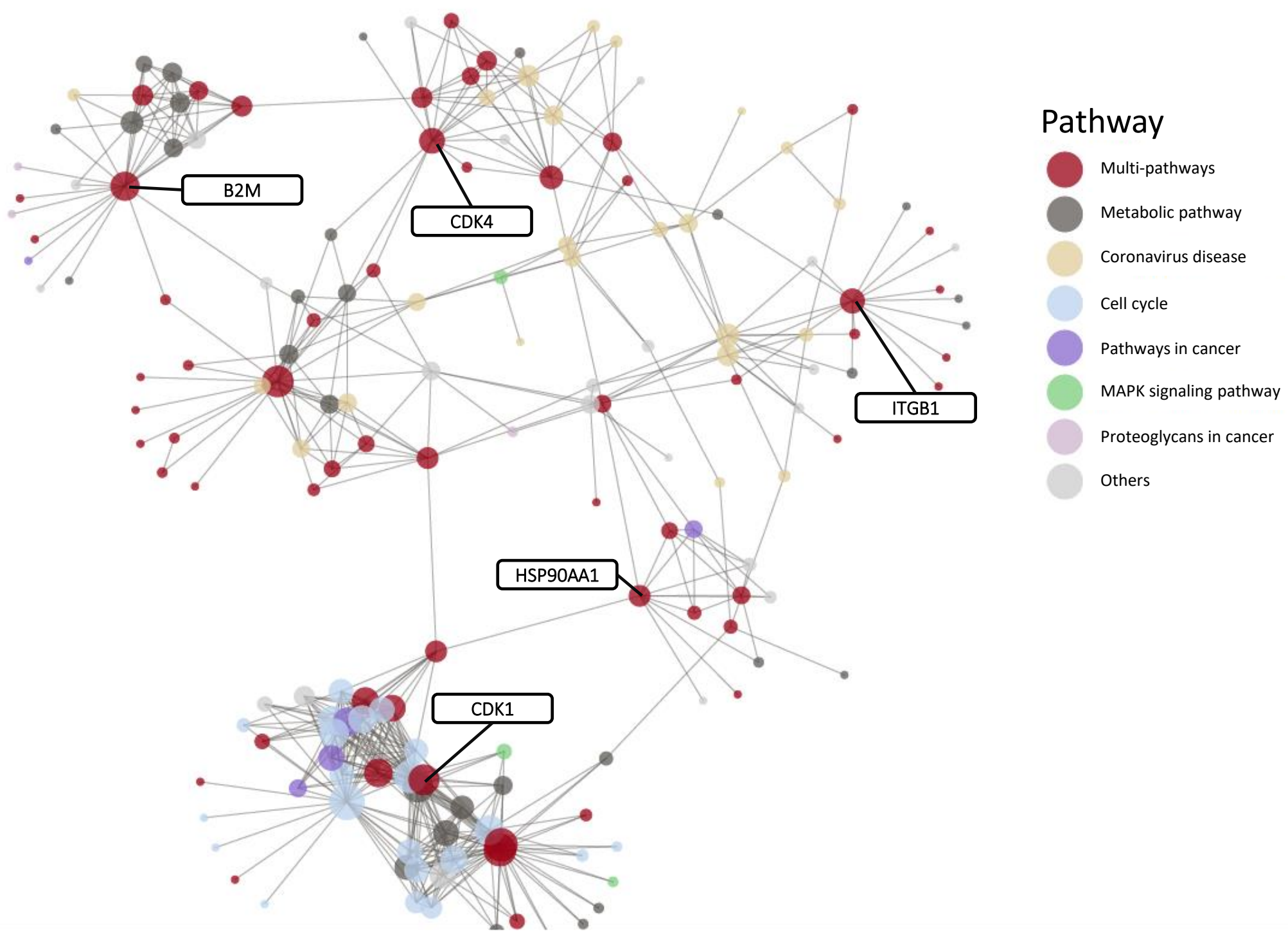

Figure A13 : Graphical representation based on the posterior mean of the inverse of the correlation matrices estimated by the model. Edge thicknesses are proportional to the latent partial correlations between genes. Values below 0.025 are not reported. Nodes are positioned using a FruchtermanReingold force-direct algorithm.

References

Asghar, U., Witkiewicz, A. K., Turner, N. C. and Knudsen, E. S. (2015) The history and future of targeting cyclin-dependent kinases in cancer therapy. *Nature Reviews Drug Discovery*, **14**, 130–146.

Legramanti, S., Durante, D. and Dunson, D. B. (2020) Bayesian cumulative shrinkage for infinite factorizations. *Biometrika*, **107**, 745–752.

Nurse, P., Masui, Y. and Hartwell, L. (1998) Understanding the cell cycle. *Nature Medicine*, **4**, 1103–1106.

Pereira, C., Gimenez-Xavier, P., Pros, E., Pajares, M. J., Moro, M., Gomez, A., Navarro, A., Condom, E., Moran, S., Gomez-Lopez, G. et al. (2017) Genomic profiling of patient-derived xenografts for lung cancer identifies b2m inactivation impairing immunorecognitionb2m inactivation in lung cancer affects immune recognition. *Clinical Cancer Research*, **23**, 3203–3213.

Polson, N. G., Scott, J. G. and Windle, J. (2013) Bayesian inference for logistic models using pólya–gamma latent variables. *Journal of the American Statistical Association*, **108**, 1339–1349.

Tian, L., Dong, X., Freytag, S., Lê Cao, K.-A., Su, S., JalalAbadi, A., Amann-Zalcenstein, D., Weber, T. S., Seidi, A., Jabbari, J. S. et al. (2019) Benchmarking single cell rna-sequencing analysis pipelines using mixture control experiments. *Nature Methods*, **16**, 479–487.

Tian, L., Su, S., Dong, X., Amann-Zalcenstein, D., Biben, C., Seidi, A., Hilton, D. J., Naik, S. H. and Ritchie, M. E. (2018) scPipe: A flexible R/Bioconductor preprocessing pipeline for single-cell RNA-sequencing data. *PLOS Computational Biology*, **14**, e1006361.

Townes, F. W., Hicks, S. C., Aryee, M. J. and Irizarry, R. A. (2019) Feature selection and dimension reduction for single-cell rna-seq based on a multinomial model. *Genome biology*, **20**, 1–16.
